# Revascularization strategies in Non-ST segment elevation myocardial infarction: the clash continues

**DOI:** 10.3389/fcvm.2025.1614843

**Published:** 2025-08-20

**Authors:** Vittorio Zuccarelli, Filippo Giunti, Mauro Chiarito, Carlo Andrea Pivato, Giulio Giuseppe Stefanini

**Affiliations:** ^1^ Department of Cardiology, Hôpital Privé Saint-Martin, Caen, France; ^2^ Department of Cardiovascular Medicine, IRCCS Humanitas Research Hospital, Milan, Italy; ^3^ Department of Biomedical Sciences, Humanitas University, Milan, Italy

**Keywords:** NSTEMI, CABG, PCI, revascularization in multivessel disease, revascularization in left main disease, revascularization in diabetic patients, hybrid coronary artery revascularization

## Abstract

For patients presenting with Non-ST-Elevation Myocardial Infarction (NSTEMI), the choice and timing of revascularization remain complex and debated. This decision is influenced by clinical factors such as hemodynamic stability, comorbidities and surgical risk profile, as well as anatomical considerations like coronary lesion complexity and feasibility of achieving complete revascularization. Randomized controlled trials directly comparing CABG and PCI in NSTEMI are limited, making evidence-based comparisons challenging. However, data suggest that while PCI is less invasive and offers rapid revascularization, CABG often achieves more comprehensive revascularization, particularly in high-risk patients with multivessel coronary artery disease, especially diabetic patients, or unprotected left main coronary artery disease. Over the last two decades, the adoption of CABG in NSTEMI has declined, driven by the advantages of PCI's minimally invasive nature and advancements in stent technology. Nevertheless, CABG remains essential in cases of complex coronary anatomy or where PCI fails to achieve adequate revascularization. Available outcome data indicate that CABG offers significant long-term benefits, including lower rates of myocardial infarction and repeat revascularization, although it is associated with an increased short-term risk of stroke, and surgical related bleeding. This review critically analyzes clinical scenarios in NSTEMI, examining the risks and benefits of CABG and PCI. It highlights the importance of individualized decision-making, guided by multidisciplinary Heart Teams, to balance procedural risks and long-term outcomes for optimal patient care

## Graphical Abstract

**Figure:**
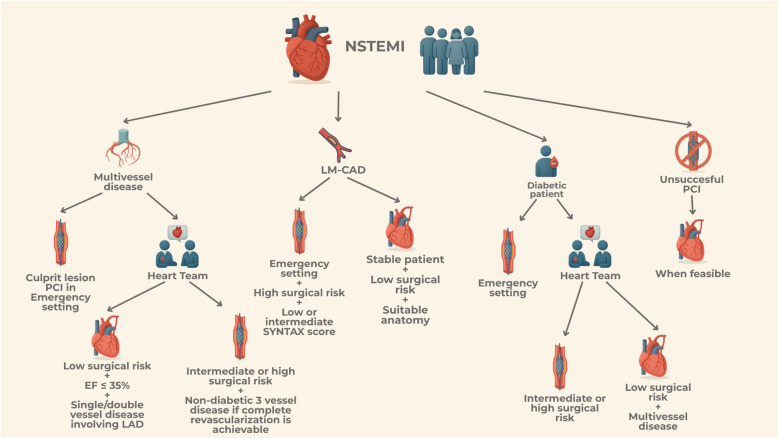
Main clinical scenarios of patients with NSTEMI.

## Introduction

The diagnosis of non-ST-segment elevation myocardial infarction (NSTEMI) in routine clinical practice is directly associated with an early invasive approach, in accordance with current European (1) and American (2) guidelines. The shortest possible delay in implementing this invasive strategy should be reserved for patients presenting with clinical signs of ongoing ischemia and those at high ischemic risk.

Over the past three decades, the use of percutaneous coronary intervention (PCI) has significantly reduced the risk of major ischemic events in these patients (3). Nonetheless, surgical revascularization remains an essential strategy, particularly in patients with left main or complex multivessel coronary artery disease, or in cases of challenging coronary anatomy that may limit the efficacy and safety of PCI.

Despite the established benefits of revascularization in NSTEMI, data comparing the long-term effectiveness of PCI vs. coronary artery bypass grafting CABG, in this clinical scenario remain limited. Urgent CABG should be considered for patients who present a coronary anatomy unsuitable or extremely challenging for PCI or, further, in case of patients needing a more complete revascularization or in case of patients presenting with cardiogenic shock (CS). Moreover, CABG is steadily recommended for patients with mechanical complications following myocardial infarction (MI) concurrently with surgical repair. In addition, CABG is indicated even after successful PCI of the culprit lesion if further bypass grafting is needed due to multivessel disease, as well as in case of incomplete or insufficient PCI, or PCI failure. Recently, American guidelines (2) have recognized that certain subsets of patients, particularly those with complex left-main or three-vessel disease, especially in the presence of diabetes, may be more appropriately managed with surgical revascularization. In these cases, the Heart-Team plays a pivotal role in tailoring the revascularization strategy, particularly in the acute setting of NSTEMI, provided the patient can be stabilized and does not require emergency revascularization. The Heart-Team should consider several factors when making this decision, including the complexity of coronary artery disease (CAD), technical feasibility of the procedure, patient's surgical risk and their potential for functional recovery and rehabilitation following CABG surgery.

Both surgical and percutaneous revascularization have advantages and disadvantages, and the choose of one over the other remains the subject of ongoing debate. Evidence from earlier studies, where NSTEMIs accounted for only a tiny fraction of the population, has shown that CABG provides improved long-term survival and a reduced incidence of major adverse cardiac events, particularly in those with complex coronary artery disease.

Conversely, the percutaneous option is much less invasive and is associated with an overall shorter hospital's length of stay, making it an appealing choice for both clinicians and patients. the choice between PCI and CABG must be carefully weighed against the patient's surgical, ischemic, and bleeding risk. Key factors to consider when selecting the appropriate revascularization strategy include the extent and complexity of coronary artery disease, the presence of mechanical complications, hemodynamic stability, surgical risk, and the patient's individual preferences.

The primary aim of this review is to examine most NSTEMI clinical scenarios, evaluating the respective risks and benefits of both CABG and PCI and to highlight the need of individualized decision making by multidisciplinary heart teams, to carefully balance procedural risk achieving long-term benefit.

## NSTE-ACS: what does the evidence say?

Strong evidence from RCTs comparing contemporary CABG and PCI in ACS patients remains limited, making direct comparisons challenging.

The 2023 European Guidelines recommend CABG for acute coronary syndromes with cardiogenic shock if PCI of the infarct related artery is not feasible or unsuccessful or in selected patients in relation to clinical status, comorbidities and anatomical complexity (1). The most recent 2025 ACC/AHA/SCAI Guidelines, recognized that certain patient subsets, such as those with complex left main disease, complex three-vessel disease and diabetes with left anterior descending artery involvement, might be optimal candidates for CABG (2). Further, the updated guidelines emphasized the central role of the Heart Team in evaluating CAD's complexity, technical feasibility and estimating patient's surgical risk.

For patients with NSTE-ACS the ideal method and timing of revascularization is still debated. The best revascularization approach depends on several factors: clinical stability, anatomical complexity, percutaneous feasibility and comorbidities that might impair procedural outcomes.

Over the past two decades, the use of CABG as the primary revascularization method after NSTE-ACS has decreased, even in high-risk patients and complex anatomical cases such as unprotected left-main (LM-CAD) and multi-vessel disease (MV-CAD). The potential benefits of more rapid revascularization with PCI, along with its limited invasive nature, may be advantageous in acute settings. On the other hand, the more complete revascularization provided by CABG might be particularly beneficial for patients with ACS (4).

It is well known that medical management of patients hospitalized with NSTE-ACS and MV-CAD is associated with worse outcomes in comparison to revascularization of any type.

Over the past twenty years, multiple randomized controlled trials —including the BEST (5), the PRECOMBAT (6) and the SYNTAX trials (7)—have evaluated the comparative efficacy of CABG and PCI across various clinical settings involving patients with left main and multivessel coronary artery disease (see Table 1). A comprehensive analysis of the effectiveness of CABG vs. PCI with drug-eluting stents in patients with NSTE-ACS and surgical anatomical patterns (LM-CAD or MV-CAD) was conducted using pooled data from these three major RCTs.

**Table 1:** PCI vs CABG in NSTE-ACS.

1st Author	YoP	n° of *p* (% of ACS)	% PCI	% CABG	FU m	I° EP	ACM	MI	S
Desperak P. (21)	2019	1,342 (100)	83,6	9,6	24 m	Higher frequency of ACS-driven revascularization in PCI group (12,7%/4,7% *p*-val = 0,031)	Similar between PCI and CABG (16.5%/20.5%)	Similar between PCI and CABG (11.1%/4,7%)	No differences in the occurrence of stroke between PCI and CABG (3%/7,1% *p*-val = 0,062)
BEST Trial; Park S.-J. (5)	2015	880 (0)	49,7	50,3	4,6 y	D, MI, TVR more frequently in the PCI groupthan in the CABG group(15.3% vs. 10.6%; hazard ratio, 1.47; 95% CI, 1.01 to 2.13; *P* = 0.04)	Similar between PCI and CABG (6.6%/5% HR 1,34 CI 0,77-2,34 *p*-val = 0,3)	Similar between PCI and CABG (4,8%/2,7% HR 1,76 CI 0,87–3,58 *p*-val = 0,11)	Similar between PCI and CABG (%2,5/ 2,9% HR 0,86. CI 0,39–1,93 *p*-val = 0,72 )
PRECOMBAT trial; Park S.-J. (6)	2011	600 (50,5)	50	50	24 m	No significant differences in incidence of D, MI, S (PCI 12.2%/CABG 8,1% HR 1,5 CI 0,9–2,52 p-val = 0,12)	Similar between PCI and CABG (2,4%/3,4% HR 0,69 CI 0,26–1,82 *p*-val = 0,45)	Similar between PCI and CABG (1,7%/1% HR 1,66 CI 0,4–6,96 *p*-val = 0,49)	Similar between PCI and CABG (0,4%/0,7% HR 0,49 CI 0,04–5,4 *p*-val = 0,56)
SYNTAX trial; Serruys P. (7)	2009	1,800 (28,4)	50,1	49,9	12 m	Lower MACCE in CABG group (12.4%) than in the PCI group (17.8%, *p*-val = 0.002)	Similar between PCI and CABG (4,4%/3,5% RR 1,24 CI 0,78–1,98 *p*-val = 0,37)	Similar between PCI and CABG (4,8%/3,3% RR 1,46 CI 0,92–2,33 *p*-val = 0,11)	Lower rates in PCI than CABG (0,6%/2,2% RR 0,25 CI 0,09–0,67 *p*-val = 0,25)
Chang M. (8)	2017	1,246 (100)	50,8	49,2	5 y	ACM, MI, S less in CABG group (13.4%) versus PCI group (18.0%) [hazard ratio (HR) 0.74; 95% CI 0.56 to 0.98; *p*-val = 0.036]	Similar between PCI and CABG (11,4%/9% HR 0.81; 95% CI 0.57 to 1.16, *p*-val = 0.248)	Lower in the CABG group (7,7%/3,9% HR 0.50; 95% CI 0.31 to 0.82, *p*-val = 0.006)	No statistical significance (2,7%/3,2% HR 1.10; 95% CI 0.56 to 2.15, *p*-val = 0.788)
Ram E. (11)	2020	5,112 (100)	84.6	15,3	10 y	No difference in MACE at 30 days between PCI and CABG (3,4% vs 3,5% *p*-val 1000) even after matching (6,9% vs 5% *p*-val = 0,342)	Long-term advantage toward CABG (*p*-val = < 0,001)	Similar between PCI and CABG ((2,3%/0,9% *p*-val = 0,018)	Similar between PCI and CABG.
Huckaby L. V. (12)	2020	2,001 (100)	26	74	3,6 y	Survival higher at 1 year (92.0 vs 81.8%; *p*-val<.001) and 5 years (80.7 vs 63.3%, *p*-val<.001) in CABG as compared to PCI.	Lower in the CABG group. (1.8 vs 7.5%, *p*-val<.001)	No significant difference (0.8% in CABG vs 1.2% in PCI, *p*-val = .479)	
Mehaffey J. H. (13)	2023	104,127 (100)	49,3	50,7	3 y	CABG had reduced unadjusted 3-year (9.9% vs 17.1%, *p*-val < .001) mortality	Lower in the CABG group (7.9% vs 14.0%, *p*-val = <.001)	Similar between PCI and CABG (2,17%/2,2% *p*-val = 0,621)	
Kakar H. (14)	2023	3,172 (100)	51,4	48,6	1 y	Multivessel PCI associated with more repeat revascularizations (OR 0.21, 95% CI 0.13 to 0.34, *p*-val < 0.00001)	No differences (OR 0.91, 95% CI 0.68 to 1.21, *p*-val = 0.51).	No differences (OR 0.78, 95% CI 0.40 to 1.51, *p*-val = 0.46)	No differences (OR 1.54, 95% CI 0.55 to 4.35, *p*-val = 0.42)
Jia S. (15)	2020	3,928 (100)	40,4	31,3	7,5 y	CABG group had a lower rate of MACCE (25.7% vs. 32.9%, *P* < 0.001)	Similar between PCI and CABG (13,2%/12,8% *p*-val = 0,761)	Lower rate in CABG (2.7% vs. 8.4%, *P* < 0.001)	Higher rate in CABG group (10.5% vs. 7.1%, *p*-val = 0.002)
Widmer R. J. (22)	2024	2,161 (100)	13,6	72,1	1 y	CABG had smaller hazards of D [[HR] 0.26, 95% confidence interval [CI] 0.19 to 0.36, *p*-val < 0.0001]	Reduction in the risk of MI within 1 year in CABG compared with PCI (HR 0.36, 95% CI 0.21 to 0.61, *p*-val < 0.0001)	Similar: *n* = 1 in the CABG cohort (0.3%), and *n* = 7 (0.5%) in the PCI group	
North Rhine-Westphalia surgical myocardial infarction registry Liakopoulos O. J. (16)	2020	2,432 (100)	10,5	100	13 d	All-cause IHM in patients with ACS undergoing surgical revascularization was 8.1%	IHM higher for patients with STEMI compared with NSTEMI and UA (12,6%/4,2%/7,6% *p*-val = < 0,001)	Similar between STEMI, NSTEMI and UA (2%/2,6%/1,9% *p*-val = 0,639)	Similar between STEMI, NSTEMI and UA (2,4%/3,1%/1,5% *p*-val = 0,121)
FAME 3 trial (5-years outcomes); Fearon W. F. (17)	2025	1,500 (587)	49,5	46	5 y	The composite of D, S, or MI occurred in 16.0% of patients in the PCI group and 14% in the CABG group (*p* = 0.27)	ACM occurred in 7.0% of patients in the PCI group and 7% in the CABG group (HR 0.99, 95% CI 0.67–1.46)	MI occurred in 8% of patients in the PCI group and 5% in the CABG group (HR 1.57, 95% CI 1.04–2.36)	S occurred in 2% of patients in the PCI group and 3% in the CABG group (HR 0.65, 95% CI 0.33–1.28)

Legend: YoP, year of publication; n° of *p* (% of ACS), number of patients (% of acute coronary syndromes); PCI, percutaneous coronary intervention; FU-m, follow up in months; I° EP, first endpoint; HR, hazard-ratio; ACM, all-cause mortality; MACE, major adverse cardiovascular events; MI, myocardial infarction; S, stroke, TVR, target-vessel revascularization; UA, unstable angina.

At 5-years, surgical revascularization has proven to be significantly superior to PCI in terms of major cardiovascular events. Moreover, the higher rate of repeat revascularization observed in the PCI group could be explained by the greater frequency of complete anatomical revascularization achieved in the CABG group, even though the studies included in the meta-analysis were based on percutaneous approaches that are now considered outdated (8). In fact, they involved either first-generation drug-eluting stents or bare-metal stents, lacked the use of intracoronary imaging, and largely reflected a period when medical therapy for controlling cardiovascular risk factors was neither as well established nor as widely adopted as it is today.

A large registry covering the last two decades of patients admitted for ACS provided valuable insights about PCI and CABG in long-term follow-up. The first study published (9), examining data from 2000–2010, found that referrals for CABG during index hospitalization, decreased over time during the study period. There was no difference in 1-year survival between PCI and CABG, despite the higher incidence of 30-days stroke in the CABG-arm. A subsequent analysis of the same registry (10), surveying trends from 2004–2016, showed that despite an increase in the percentage of ACSs treated with MV-PCI over time, compared to CABG, 1-year mortality rate remained equivalent. During the 12-year follow-up period, both groups showed overall improvements in 30-day major cardiac events, mortality and reinfarction rates. However, for what pertained patients admitted with NSTE and enrolled in the registry (11), unadjusted mortality at 10-years follow-up was reduced in the CABG group, a trend that persisted after propensity matching. The protective effect of CABG emerged after the third year of follow-up, in line with what emerged in several cohort studies with longer follow-up, possibly emphasizing the importance of completeness of revascularization in terms of mortality and hard endpoints.

Several recent studies showed encouraging results of surgical revascularization in NSTE-ACS.

A recent U.S. registry reported 5-year outcomes for 2,000 patients with NSTE-ACS and multivessel disease. CABG was linked to better survival at both 1 and 5 years compared to those who received multivessel PCI and this survival advantage remained significant even after adjusting for complete revascularization. Additionally, CABG was associated with a lower adjusted risk of major adverse cardiovascular events and hospital readmissions (12). Even recent (2018–2020) real-world data, based on a large number of patients (around 100,000), confirmed that surgical revascularization offers significant benefits in terms of lower in-hospital mortality, fewer hospital readmissions at three years, fewer coronary reinterventions and improved 3-year survival (13). The advantage in terms of reducing unplanned revascularizations is a well-established finding (14, 15).

The completeness of revascularization achieved with CABG seems to play a key role in improving overall survival and reducing the risk of major cardiovascular events, both in the short and long term.

Contemporary data from the SWEDEHEART established that surgical revascularization had lower risks of mortality, MI, hospitalization for heart failure and unplanned revascularization. These benefits emerged predominantly in certain high-risk subgroups, as patients with reduced ejection fraction, diabetes, left main or three vessel disease. Unsurprisingly, these subgroups represent the population in which the evidence supporting the benefits of CABG is strongest. However, the long-term survival advantage of CABG diminished in patients with shorter life expectancy, with the greatest benefit observed in those under 70 years of age who had left main disease or left ventricular dysfunction (3).

Despite this encouraging results, everyday clinical practice indicates that myocardial revascularization in NSTEMI continues to be associated with significant in-hospital mortality, particularly when emergency CABG is performed, which is linked to poorer outcomes. This can be partially explained by the fact that, in clinical routine as reflected in the SWEDEHEART, patients often have multiple comorbidities, high incidence of three vessel-CAD and LM-CAD. Additionally, most patients in the registry underwent off-pump surgery, which is common in everyday practice, and only a very small number received multiple arterial grafts (16). From an interventional point of view, although several studies have noted a higher incidence of repeat revascularization in patients treated with PCI, this may be attributed to the use of early-generation stents, which had higher restenosis rates compared to contemporary devices. Further, the higher rate of incomplete revascularization, which significantly impacts long-term clinical outcomes in patients with MV-CAD, may be an outdated issue. Over the past two decades, advancements in PCI tools and techniques have significantly improved and may now be comparable to multivessel grafts.

Promising results for PCI in the management of three-vessel CAD without left main involvement have emerged from the most recent 5-year fu of the FAME-3 (17). This trial compared fractional flow reserve (FFR)-guided PCI using current-generation zotarolimus-eluting stents with CABG. Despite including only 40% ca patients hospitalized for ACS in both arms, at five years, there was no significant difference between the two groups in the composite endpoint of death, stroke, or myocardial infarction. Similarly, rates of death and stroke individually were comparable between the groups; however, the PCI group demonstrated a higher incidence of myocardial infarction and repeat revascularization.

In contrast to earlier studies, the FAME-3 trial reported lower absolute rates of death, stroke, or MI at 5 years in both treatment arms and no evidence of progressive divergence in outcomes over time favoring CABG. These results may be narrowing the historical gap in outcomes of PCI compared with CABG. This improvement is largely attributed to the evolution of PCI techniques. In fact, the use of contemporary drug-eluting stents correlates with significantly less rates of stent thrombosis, restenosis and long-term adverse events. In addition, routine use of FFR to guide PCI has led to targeted revascularization of ischemia-producing lesions and reducing the risk of treating functionally non-ischemic stenosis, further reducing the long-term complications linked to multi-stent PCIs.

Interestingly, despite the higher incidence of MI in the PCI group in the SWEEDHEART, there was no parallel increase on all-cause mortality, suggesting that MI after PCI may be a suboptimal surrogate endpoint for long-term survival. Further, the established trade-offs between the two strategies are confirmed from this contemporary data: CABG means longer initial hospital stays, increased perioperative complications and a higher risk of early rehospitalization, whereas PCI carries a greater long-term risk of repeat revascularization.

Despite being a multicenter randomized trial, FAME-3 revealed several limitations closely reflecting real world scenarios. In the CABG arm, only a quarter of patients received multiple arterial grafts, despite guideline recommendations while, in the PCI arm, intravascular imaging was utilized in only one procedure out of ten. These findings underscore critical areas where current clinical practice can be enhanced in both revascularization strategies.

The 2021 ACC/AHA/SCAI American guidelines had challenged the role of CABG in complex coronary anatomy downgrading its level of recommendation, despite available data showed a significant survival benefit of surgical revascularization. As a result, that guidelines had been formally rejected by the major cardiac surgery societies in North America (18), followed by those across Europe (19) and South America (20). Contrarywise the updated 2025 Guidelines (2), recognized the pivotal role of surgery in patients with complex left-main and three-vessel coronary artery disease and diabetic patients.

In the absence of large, contemporary randomized trials directly comparing PCI and CABG specifically in NSTEMI patients, clinical decision-making must remain patient-centered, integrating anatomical, procedural, and comorbidity-related factors. Until such data emerge, the accumulated evidence continues to support CABG as a robust and effective strategy in appropriately selected patients with complex coronary artery disease and recent guideline shifts that de-emphasize its role may warrant critical reassessment.

## Left-main CAD: a tug of war between surgeons and interventional cardiologists

Left main coronary artery disease (LMCAD) is recognized as a critical category associated with the highest mortality in literature both in stable and primarily in unstable patients. Traditionally, CABG has been the preferred treatment, especially in diabetic patients, but both Europeans (1) and American (2) guidelines acknowledge that PCI with drug-eluting stents can be considered for patients with low-to-intermediate anatomical complexity, without diabetes (see Table 2). Long-term survival outcomes remain uncertain, as the trials comparing these strategies are challenging to conduct.

**Table 2:** PCI vs CABG in LM-CAD.

1st Author.	YoP	n° of *p* (% of ACS)	% PCI	% CABG	FU m	I° EP	ACM	MI	S
Palmerini T. (23)	2017	4,686	50	50	39 m	PCI had lower 30-day rates of stroke (OR 0.36, 95% CI 0.16–0.82,*P* = .007), lower 30-day all-cause death or MI (OR 0.69, 95% CI 0.49–0.98,*P* = .04), and lower 30-day rates of ACM, MI, or S (OR 0.64, 95% CI 0.45–0.90, *P* = .01) compared with CABG.	no significant differences (HR 0.99, 95% CI 0.76–1.30) in late outcomes.	no significant differences (HR 1.33, 95% CI 0.84–2.11) in late outcomes.	no significant differences (HR 0.71, 95% CI 0.34–1.49) in late outcomes.
SYNTAX trial (10-year outcomes); Thuijs D. J. F. M. (24)	2019	1,800 (28,5)	50	50	10 y	No significant difference in all-cause death between PCI and CABG (28%/24% HR 1,19 95% CI 0,99–1,43 *p*-val = 0·066)			
EXCEL trial, (Five-Year outcomes) Stone G. W (25).	2019	1,905	60	40	5 y	D, S, MI: 22.0% in the PCI group and in 19.2% in the CABG group [difference, 2.8 percentage points; 95% confidence interval (CI), −0.9 to 6.5; *P* = 0.13]	CABG better than PCI (in 13.0% vs. 9.9%; difference, 3.1 percentage points; 95% CI, 0.2 to 6.1)	Similar between CABG and PCI (10.6% and 9.1%; difference, 1.4 percentage points; 95% CI, −1.3 to 4.2)	Similar between PCI and CABG (2.9% and 3.7%; difference, −0.8 percentage points; 95% CI, −2.4 to 0.9)
PRECOMBAT Trial (10-Year Outcomes) Park D. W. (26)	2020	600 (45)	50	50	10 y	D, MI, S, or ischemia-driven TVR occurred in 29.8% of the PCI group and in 24.7% of the CABG group [hazard ratio [HR] with PCI vs CABG, 1.25 [95% CI, 0.93–1.69]]	Similar between CABG and PCI [14.5% vs 13.8%; HR 1.13 (95% CI, 0.75–1.70)]	Similar between PCI and CABG (3,2%/2,8% HR 0,76 CI 0,32–1,82)	Similar between PCI and CABG (1,9%/2,2% HR 0,71 CI0,22–2,23)
NOBLE trial (5-year outcomes); Holm N. R. (27)	2020	1,201 (26)	50	50	5 y	MACE occurred less frequent in CABG than PCI (28%/19%, HR 1·58 95% CI 1·24–2·01 *p* = 0·0002)	Similar between CPCI and CABG (9,4%/8,7% HR 1,08 CI 0,74–1,59 *p*-val = 0,68)	CABG better than PCI (2,7%/7,6% HR 2,99 CI 1,66–5,39 *p*-val = 0,0002)	Similar between PCI and CABG (3,8%/2,2% HR 1,75 CI 0,86–3,55 *p*-val = 0,1109)
Gaba P. (28)	2023	4,394 (33)	2,928	2,197	10 y	At 30 days, patients with ACS had higher rates of ACM compared with those without ACS (1.9% vs 0.5%; HR, 3.40; 95% CI, 1.81–6.37; *P* < .001)	No significant differences (ACS: HR, 0.97; 95% CI, 0.74–1.27; not ACS: HR, 1.18; 95% CI, 0.96–1.44; *P* = .27 for interaction)	ACS patients undergoing PCI vs CABG (HR, 1.74; 95% CI, 1.09–2.77) higher in CCS patiens undergoing PCI vs CABG (HR, 3.03; 95% CI, 1.94–4.72; *P* = .09 for interaction)	at 5 years, no difference in PCI and CABG (2,15%/1,8% *p*-val 0,35)
Kirov H. (30)	2022	48,891 (100)			No significant difference in long-term mortality (IRR 0.93, 95% CI 0.70; 1.23, *p*-val = 0.83)	No significant difference (IRR 0.93, 95% CI 0.70; 1.23, *p*-val = 0.83)	No significant difference (IRR 0.96, 95% CI 0.50; 1.84, *p*-val = 0.61)	No significant differences (IRR 0.96, 95% CI 0.72; 1.28, *p*-val = 0.81)	
Moussal D. (31)	2020	5,100,394 (77,1)	100	0	2 d	No significant differences in in-hospital outcomes.	No differences between ISR PCI and non-ISR PCI (0,5%/0,6%)	No differences between ISR PCI and non-ISR PCI (1,6%/1,8%)	No differences between ISR PCI and non-ISR PCI (0,2%/0,2%)

Legend: YoP, year of publication, n° of *p* (% of ACS), number of patients (% of Acute coronary syndromes); PCI, percutaneous coronary intervention; FU-m, follow up in months; I° EP, first endpoint; MACE, major adverse cardiovascular events, ACM, all-cause mortality; MI, myocardial infarction; S, stroke; TVR, target vessel revascularization.

An outdated metanalysis of individual patient data, showed that, in patients undergoing revascularization for unprotected-LM CAD, PCI and CABG were associated with similar mortality rates at a median follow-up of 3 years. However, an interaction effect indicated relatively lower mortality with PCI in patients with a low SYNTAX score and relatively lower mortality with CABG in those with a high SYNTAX score. Both procedures resulted in comparable long-term composite rates of death, myocardial infarction, or stroke, with PCI offering an early safety advantage and CABG showing greater durability (23). However, no data on mortality in relation to the completeness of revascularization were available and the median follow-up period was too short to determine any long-term differences between the two revascularization strategies.

During the last twenty years, four randomized controlled trials were powered enough to properly investigate clinical differences and compare the outcomes of patients undergoing PCI vs. CABG in stable and unstable clinical scenarios. These trials included: the SYNTAX trial (24), the EXCEL trial (25), the PRECOMBAT trial (26) and the NOBLE trial (27).

A contemporary patient-level analysis from the abovementioned RCTs including aroung 4,400 patients with LMCAD, revealed 1.0% absolute risk difference (<0.2% per year) between PCI and CABG in 5-year all-cause mortality. Notwithstanding, the excess of mortality observed was primarily non-cardiovascular and there was no progressive divergence in cardiovascular mortality over time. As previously reported in many studies, PCI-treated patients had higher rates of spontaneous MI and repeated revascularization at 5-years. Stroke rate, instead, was initially lower with PCI within the first-year post-randomization, but this difference was not significant by 5 years.

One important point in favor of the surgical strategy, as highlighted in this patient-level analysis of the most prominent RCTs on this topic, is the anatomical complexity of the patients included. In fact, three-quarters of the patients had a SYNTAX score in the low to intermediate range (<32 in 75% of patients). This is largely because some of the trials included in the analysis, such as the EXCEL tri, excluded patients with a high SYNTAX score. Therefore, the observed differences may have been attenuated by the exclusion of this higher-risk subgroup. It is likely that the advantage of surgical revascularization would have been even greater, given the well-documented higher long-term complication rates associated with PCI in patients with high anatomical complexity.

It is important to emphasize that these four most significant RCTs span a wide period and reflect the evolution of stent technology and PCI techniques over the past two decades. Additionally, the selection criteria varied across trials due to changes in the definition of ACS. Despite being conducted over more than a decade, the results remain relatively consistent.

A subsequent analysis (28), from the same pooled database, of patients with left main involvement revealed that those presenting with ACS had higher rates of early cardiovascular death and spontaneous MI throughout the follow-up period, in comparison to stable CAD. These patients were characterized by having higher SYNTAX scores and comorbidities, in particular diabetes. However, contrary to what might be expected when considering patients in an acute setting characterized by high anatomical complexity (high SYNTAX score), diabetes and impaired ventricular ejection fraction; no substantial difference emerged between percutaneous and surgical revascularization in terms of 5- and 10-year survival. Although, the risk of spontaneous MI and repeated revascularization remained consistently higher during follow-up in patients treated with PCI. In light of this, the advantage of CABG in patients with greater anatomical complexity may lie in the unexamined benefit of complete anatomical revascularization. Indeed, it remains unknown how many patients in either group achieved complete revascularization and how this may have influenced the outcomes (29).

Several analyses emphasized that among ACS patients who underwent PCI, the majority faced long-term issues such as target lesion revascularization and repeated revascularization, with 25% experiencing spontaneous MI (30, 31). Given the increased risk for recurrent events throughout the coronary tree after ACS and the vessel-level protection offered by graft, a greater relative benefit for CABG compared to PCI for spontaneous MI and repeat revascularization in the ACS was expected (32) and was confirmed in this analysis.

It is important to note that, as a meta-analysis based on RCTs with strict selection criteria, the patients included did not represent the full spectrum of ACS presentations. Specifically, the enrolled patients were stable and did not present cardiogenic shock, ongoing infarction, nor required emergent revascularization.

In the context of ACS requiring emergent LM revascularization, a multicenter retrospective cohort study found that patients undergoing emergency PCI were older and had a higher prevalence of chronic kidney disease, lower ejection fraction and higher surgical risk compared to CABG patients, who had high SYNTAX scores and more frequent multivessel disease. In emergent revascularization, PCI was associated with significantly lower hard cardiovascular endpoints and in-hospital mortality than CABG. Additionally, PCI was linked to reduced hospital mortality in emergent patients with intermediate and high EuroSCORE, as well as those with low and intermediate SYNTAX scores. At a median follow-up of 20 months, emergency PCI showed lower cardiovascular events compared to CABG, with no significant difference in all-cause mortality (33).

In conclusion, as clinically accepted, CABG is generally recommended for ACS patients with LMCAD who do not require emergent revascularization, have low surgical risk, and possess complex anatomy. Conversely, PCI may be advantageous for emergent LMCA revascularization and could be preferred in non-emergent cases for patients with intermediate or high surgical risk and low to intermediate SYNTAX scores.

## Unsuccessful PCI in ACS patients

The management of a failed PCI in acute coronary syndromes remains a largely unaddressed and underexplored area. Current literature provides limited support on this topic, leaving a significant gap in clinical practice.

An important European registry focused on the risk assessment and surgical outcomes of patients referred to CABG surgery after a prior PCI procedure, either following a successful PCI of the culprit lesion with an additional indication for CABG surgery or where PCI was incomplete, insufficient, or failed. The study highlighted that emergency CABG after PCI is associated with substantial intra-hospital mortality and major cardiovascular events. Further, by differentiating PCI subgroups based on urgency, it emerged that CABG within 24 h of PCI, as well as failed PCI, are associated with considerable perioperative risk and cardiogenic shock, leading to increased morbidity and mortality following surgery (34). Unsuccessful, complicated or failed PCI were major determinants of mortality in patients who underwent CABG after PCI, particularly in the context of primary PCIs (35).

Research has shown that elective CABG surgery outcomes in terms of mortality rate and hard endpoints can be compromised by prior PCI procedures, affecting both short- and long-term prognoses (36–38). In the current era of PCI treatment, 15%–30% of PCI-treated patients with coronary artery disease will require additional coronary revascularization, with nearly 20% being referred to CABG surgery at some point after stenting. The number of “stent-loaded” patients is increasing and will likely continue to grow. While the situation for patients with ACS differs from elective coronary artery disease treatment, those with severe or end-stage coronary artery disease and acute myocardial infarction primarily treated with PCI may still need CABG surgery.

Everyday clinical practice confirms these findings. Indeed, a non negligeable proportion of NSTEMI are referred to CABG after initial coronary angiography without any PCI-attempt. This is particularly true for patients with severe multivessel or left main-stem lesions, particularly in proximal segments without severe involvement in distal coronary arteries, where PCI could lead to suboptimal results or angiographic fails.

Among NSTEMI patients where PCI of the culprit lesion has been done, the risk of subsequent surgical revascularization is not decreased. Instead, both mortality and major cardiovascular events are significantly increased in this subgroup of patients. The registry mentioned above indicates that one out ten patients presenting with ACS experienced unsuccessful PCI, whether elective or emergent. This highlighted that ACS patients, especially those with STEMI and cardiogenic shock, face the highest perioperative risk for in-hospital mortality and cardiovascular events during emergency CABG surgery. Despite a potential decline in the rate of failed PCI, the rate of patients requiring CABG remains currently high (34).

With the growing number of patients with complex coronary anatomy and a prior stent burden, the intersection between PCI failure and emergent surgical referral remains an underdefined clinical situation, characterized by heightened perioperative risk, limited predictive tools and less favorable prognosis. This underscores an urgent need for robust prospective investigations to refine decision-making algorithms and optimize the timing and modality of revascularization.

## PCI vs. CABG in diabetic patients

Targeted analyses and robust evidence guiding the choice of the optimal revascularization strategy in diabetic patients presenting in acute settings are currently limited.

The landmark Freedom-trial (39) was pivotal in assessing coronary revascularization in diabetic patients with multivessel disease, demonstrating CABG's superiority over PCI in terms of survival and nonfatal MI at 5 years. This survival benefit was also evident in an extended follow-up cohort (mean follow-up, 7.5 years) (40). Consistent with prior studies, the incidence of stroke was significantly elevated in patients undergoing CABG compared to those receiving alternative revascularization strategies. Notably, patients with LM-CAD were excluded from this trial and the study population included both stable and ACS patients.

Subsequent studies evaluated the translation of these results to everyday clinical practice. A large Canadian study highlighted that patients with NSTEMI undergoing had a lower incidence of composite major adverse coronary and cerebral events, compared to PCI, despite having an higher risk of stroke. Despite this, as in the Freedom-trial, patients with LM-CAD and patients in cardiogenic shock were excluded (39).

A sub-analysis of the ACUITY (41), focusing exclusively on diabetic patients with MV-CAD and LAD-involvement, revealed that PCI was associated with lower rates of major bleeding and acute kidney injury compared to CABG, despite showing higher rates of unplanned revascularization at 1 year. Notwithstanding, no significant differences arose in terms of mortality, MI or stroke.

Subsequently, a wide observational study involving diabetic patients with NSTEMI and MV-CAD, emphasized that over a 3-year follow-up period, CABG was linked to improved overall-mortality and lower rates of major events, new-onset MI and stroke, compared to PCI (39).

It is important to emphasize that outdated clinical trials often fail to capture the impact of contemporary medical therapy and interventional advancements (see Table 3).

**Table 3:** PCI vs CABG in diabetic patients with NSTEMI.

1st Author	YoP	n° of *p* (% of ACS)	% PCI	% CABG	FU m	I° EP	ACM	MI	S
FREEDOM trial; K. (39)	2017	4,819 (63,2)	60	40	3,3 y	Among ACS patients, OR for MACE favored CABG (adjusted OR: 0.49; 95% CI: 0.34 to 0.71), whereas among CCS patients MACCE did not vary on the basis of revascularization strategy (OR: 1.46; 95% CI: 0.71 to 3.01).	Lower in CABG compared with PCI (10,3%/19% HR: 0.48; 95% CI: 0.39 to 0.59 *p*-val = <0,01)	Lower in CABG compared with PCI (6,8%/15,5% HR: 0.40; 95% CI: 0.31 to 0.51 *p*-val = <0,01)	Similar (4,1%/5,1% HR: 0.80; 95% CI: 0.56 to 1.15 *p*-val = 0,23)
The FREEDOM Follow-On Study. Farkouh M. E. (40)	2,019	943 (28,5)	50	50	80,4 m	CABG better than PCI in ACM [24.3%/18.3% unadjusted hazard ratio [HR]: 1.36; 95% confidence interval [CI]: 1.07 to 1.74; *p* = 0.01]	CABG better than PCI in ACM [24.3%/18.3% unadjusted hazard ratio [HR]: 1.36; 95% confidence interval [CI]: 1.07 to 1.74; *p* = 0.01]	Similar between PCI and CABG (4.7%/4.0%)	Similar between PCI and CABG (2.3% vs. 1.5%)
Analysis from the ACUITY trial Ben-Gal Y. (41)	2015	1,772 (100)	75,2	23,8	1 y	Patients undergoing PCI compared with CABG had significantly lower unadjusted 1-month rates of mortality (1.6% versus 4.7%; *P* < 0.0003), MI, major bleeding, and acute renal injury, but underwent more repeat revascularization procedures.	No significant difference between the PCI and CABG groups (6.3% versus 7.2%, respectively; *P*=0.23)	Q-wave MI occurred more frequently in the CABG group [11,7%/7,1% HR 1.57 (1.11–2.22) *p*-val = 0,01]	Similar between PCI and CABG (0,7%/0,2% *p*-val = 0,15)
Bangalore S. (42)	2015	8,096	50	50	4 y	EES showed lower risk of death [23 [0.57%] versus 45 [1.11%] events; HR=0.58; 95% CI, 0.34–0.98; *P*=0.04] and stroke [10 [0.25%] versus 57 [1.41%] events; HR=0.14; 95% CI, 0.06–0.30; *P* < 0.0001] but higher risk of MI [18 [0.44%] versus 11 [0.27%] events; HR=2.44; 95% CI, 1.13–5.31; *P*=0.02] compared with CABG.	Similar risk [425 [10.50%] versus 414 [10.23%] events; HR=1.12; 95% CI, 0.96–1.30; *P*=0.16].	Higher risk of MI in EES [260 [6.42%] versus 166 [4.10%] events; HR=1.64; 95% CI, 1.32–2.04; *P* < 0.0001] when compared with CABG	Lower risk of stroke in EES [118 [2.92%] versus 157 [3.88%] events; HR=0.76; 95% CI, 0.58–0.99; *P*=0.04] when compared with CABG

Legend: YoP, year of publication; n° of p (% of ACS), number of patients (% of Acute coronary syndromes); PCI, percutaneous coronary intervention; FU-m, follow up in months; I° EP, first endpoint; ACM, all-cause mortality; MACE, major adverse cardiovascular events; MI, myocardial infarction; S, stroke.

A large-scale analysis of more than 16,000 diabetic patients with MV-CAD who undergoing PCI with second-generation drug-eluting stents proved superior short-term outcomes (30-day mortality and stroke rates) compared to CABG. In the long term, survival curves between the two strategies were overlapping; however, PCI was linked to a higher incidence of unplanned revascularization and a lower risk of stroke (42). These findings underscore the clinical relevance of modern stent platforms and advanced PCI techniques, such as intravascular imaging and stent optimization, which have remarkably influenced prognostic outcomes and hard endpoints in routine clinical practice.

From the interventional point of view, previous trials were conducted using outdated stent platforms associated with higher rates of stent thrombosis and restenosis. Dated PCI techniques and tools may have resulted in incomplete revascularization and suboptimal results, leading to an overestimation of adverse events ad worse long-term outcomes.

Recent clinical trials, large registries and meta-analyses have consistently shown that newer-generation DES offer superior efficacy and safety compared to first-generation DES or bare-metal stents, reducing restenosis rates, stent thrombosis, MI and death, even in patients with diabetes (40, 43). Furthermore, advances in PCI equipment and techniques, including high success rates for complex procedures, have improved the potential for complete revascularization with PCI without a worsening of long-term outcomes.

From a surgical perspective, previous trials rarely employed techniques such as pan-arterial grafts, minimally invasive CABG or off-pump surgery, even though these are infrequently used in every-day practice. Further, a common opinion shared by cardiac surgeons is that identifying a true culprit lesion in diabetic patients with ACS is sometimes difficult, so the most complete revascularization offered by CABG may play a key role in protecting patients against recurrent ischemic events.

Despite the well-known superiority of these techniques over conventional surgery in several clinical scenarios, this has not been demonstrated in well-powered randomized trials.

To sum up, current evidence in diabetic patients undergoing revascularization highlight the need of a tailored approach in acute settings. This approach must take into consideration patients' comorbidities, coronary anatomy, technical feasibility and the prospect of achieving complete revascularization. Such decisions should be made collaboratively within a Heart Team when they can be delayed and comprehensively discussed.

## Hybrid revascularization in NSTEMI

Hybrid coronary revascularization (HCR) is defined as a combined revascularization involving left internal mammary artery (LIMA) graft to LAD and PCI of non-LAD (left anterior descending artery) vessels for the treatment of MVD (44). HCR combines the durability of the LIMA graft to the decreased invasiveness of PCI, often in a staged fashion. HCR may offer the advantages of both procedures while attempting to minimize risks.

The rationale for HCR lies in the well-established survival benefit conferred by LIMA-to-LAD grafts, associated with 95% patency at 10 years and 88% at 15 years (45), and the use of last generation stent platforms associate with lower rates of stent restenosis/ thrombosis compared with venous graft occlusion (46).

The ideal candidate for HCR is a patient with multivessel CAD and at least one of the following anatomical characteristics: proximal complex LAD lesion with optimal distal anatomy amenable to LIMA-grafting and non-LAD lesions amenable to PCI or complex distal LM lesions where circumflex artery is amenable for PCI.

HCR results as well appealing for high-risk patients with a contraindication for cardiopulmonary bypass surgery via midline sternotomy, including those with a high risk of sternal wound infection, such as diabetics and severely obese (47).

From a surgical standpoint, the LIMA-to-LAD graft can be performed using various approaches, including the open sternotomy off-pump coronary artery bypass (OPCAB), the sternal-sparing minimally invasive direct coronary artery bypass (MIDCAB), and the more recent robotic-assisted totally endoscopic technique. It should be emphasized that contemporary HCR protocols overtly exclude the use of cardiopulmonary bypass.

All sternal-sparing HCR techniques, partly due to be off-pump, have been associated with reductions in neurological events, bleeding severity, infection rates, duration of mechanical ventilation in the ICU and overall hospital length of stay compared with conventional CABG (48).

Among matched patients with comparable anatomical complexity and statistically similar preoperative risk profiles, those who underwent HCR with an off-pump sternal-sparing LIMA-to-LAD graft followed by PCI demonstrated a similar incidence of cardiovascular events at 3-year follow-up compared to patients treated with multivessel CABG, although NSTEMI cases comprised only a negligible proportion. Further, the CABG group had a higher incidence of blood transfusion, while the HCR group exhibited a greater need for repeat revascularization (49). Notably, patients with severe comorbidities and complex coronary anatomy, particularly those unsuitable for PCI on the LAD, may benefit more from HCR due to the avoidance of aortic manipulation and cardiopulmonary bypass.

For non-LAD vessels, drug-eluting stents are preferred over saphenous vein grafts (SVGs) which still remain the most used conduits for non-LAD bypass globally, despite surgical societies recommendation of pan-arterial revascularization (50). Indeed, SVGs have ephemeral durability, with approximately 45% failing within 12–18 months (51) and >70% by 15 years (52). In contrast, contemporary stents offer long-term patency rates of >96% (53).

Regarding HCR timing strategies, three approaches are currently available: simultaneous CABG and PCI (one-stop HCR), a CABG-first strategy, and a PCI-first strategy.

One-stop HCR is performed in a hybrid suite, starting with surgery first, followed by PCI. A significant advantage is the immediate assessment of the LIMA-LAD anastomosis by angiography (54). Thus, any major issue with the graft can be addressed. Moreover, the non-LAD PCI is performed with LAD territory already protected (55). However, hybrid suites are costly and scares worldwide. A propensity-matched analysis demonstrated that, following stratification by surgical risk and coronary anatomy using the EuroSCORE and SYNTAX score respectively, the cumulative incidence of major cardiovascular events was lower among high-risk patients with complex coronary anatomy who underwent one-stop HCR, compared to those treated with either multivessel PCI or CABG (56).

The CABG-first approach is the most commonly employed. Indeed, US data indicate that among 775,000 patients with multivessel CAD, only 0.2% underwent hybrid revascularization. Among these, the majority (69%) were treated using a CABG-first strategy (57). The advantage of this approach is the possibility to assess the quality of LIMA to LAD anastomosis by angiography prior to PCI, possibly addressing potential issues. After the LIMA graft is assessed, stent implantation of the remaining diseased segments is performed.

On the other hand, the PCI-first approach is way too common in ACS-NSTEMI when the culprit lesion is a non-LAD lesion. Stenting of the non-LAD arteries is performed first and the LIMA to LAD is scheduled, often after 30 days, to leave a safe 30-days protection under DAPT (58).

A major challenge associated with HCR is the management of antiplatelet therapy, particularly in balancing the risk of perioperative bleeding with the prevention of stent thrombosis in the acute setting.

In most registries reporting outcomes of one-stop HCR, patients underwent the LIMA-to-LAD graft under aspirin monotherapy, with the second antiplatelet agent generally introduced immediately after PCI (59, 60). Conversely, in registries evaluating the CABG-first approach, where ACS cases represented only a minimal proportion, surgery was performed on aspirin alone, and the second antiplatelet agent was added postoperatively only if no bleeding complications occurred (61, 62).

In the “PCI-first” approach, DAPT is typically initiated prior to PCI and continued without interruption during subsequent CABG (49). Recently, the use of cangrelor has emerged as a potential strategy to safely interrupt DAPT even during the high-risk ischemic period following stent placement (<30 days). This approach allows for temporary discontinuation of oral P2Y12 inhibitors exclusively during the perioperative phase, with prompt resumption of DAPT after surgery.

All the strategies to manage antiplatelet and anticoagulant therapy perioperatively, as well as DAPT following stent implantation, have been underexplored and applied to a limited number of carefully selected patients, introducing significant selection bias despite the favorable outcomes reported (56, 60)

The exact timing and dose of antiplatelet therapy during the CABG-first and simultaneous HCR are not clearly described, highlighting the need for more robust clinical guidance.

Although no randomized trials currently support HCR compared to either CABG or multivessel PCI, encouraging evidence from prospective cohort and observational studies suggests potential benefits of the hybrid revascularization. However, these studies typically include CCS, especially the ones comparing HCR and surgery, with ACS patients frequently accounting for less than 30% of the study population. Notably, all comparisons of HCR vs. CABG published to date have suffered from inadequate sample size.

A prospective multicenter observational pilot study investigated the characteristics and outcomes of patients with hybrid-eligible coronary anatomy, defined as a LAD lesion plus at least 1 other non-LAD lesion, on whom ca 18% of NSTEMI, undergoing either HCR, with all the aforementioned surgical approaches or multivessel PCI. The study showed no significant difference in major cardiovascular events at 18 months between groups after adjusting for baseline risk, underscoring the need for a randomized trial to directly compare the effectiveness of these two revascularization strategies (63). The only RCT available comparing HCR to standard CABG, the POL-MIDES study, enrolled only CCS. Notwithstanding, it demonstrated the actual feasibility ans safety of HCR with a tax of conversion to CABG only of 6% and proved no difference in terms of mortality and major events between the two groups at 1-yr follow up (54).

In conclusion, HCR represents an emerging revascularization strategy that warrants thorough investigation. By offering the potential to reduce bleeding, ventilator time, and hospital length of stay compared with conventional CABG; while preserving the durability and survival benefits of the LIMA-to-LAD graft, HCR provides an optimal integration of surgical and percutaneous techniques, especially in the acute setting of NSTEMI. This synergistic approach may be a valuable option for patients with multivessel CAD. However, unresolved issues, such as the management of anticoagulation, antiplatelet therapy, and surgery-related bleeding, must be adequately addressed in future studies.

## Peri-procedural myocardial infarction in NSTEMI

Periprocedural myocardial infarction (pMI) has traditionally been included in randomized controlled trials as a primary composite outcome, accepted as a surrogate for mortality (64). However, its definition and prognostic importance have been controversial. Advances in cardiac imaging and lab essays have increased pMI detection, but whether pMI considerably affects survival remains debated.

For stable patients undergoing elective revascularization, pMI during PCI and CABG has multiple causes. During PCI, lesion preparation commonly contributes to myocardial damage. Common mechanisms include side-branch occlusion (ca 60%) and distal coronary embolization (ca 15%) (65, 66).

In CABG, early graft failure is the primary cause of pMI (up to 12% of cases, though only 3% are clinically evident) (67). Mechanisms most involved are graft kinking/thrombosis, anastomotic stenosis, or global hypoperfusion (68). Less frequent causes involve technical aspects like cardiac manipulation, cardiopulmonary bypass, reperfusion injury, and cardioplegia (69).

The definition and prognostic role of pMI and type 4a myocardial infarction, as per the Fourth Universal Definition of Myocardial Infarction (UDMI) (70), are well established in patients with chronic coronary syndromes undergoing PCI with non-elevated baseline cTn levels (71).

Conversely, their incidence, interpretation, and prognostic relevance in NSTEMI patients remain unknown, as per the recent ESC/EAPCI Working Group consensus (71).

According to the Fourth UDMI, for patients with stable or falling baseline cTnI, post-PCI cTnI increase more than 20% with an absolute postprocedural value ≥5 times the 99th percentile upper reference limit (URL) defines pMI (70).

Type 4a MI requires pMI plus one of the following: (1) new ischemic ECG changes, (2) new pathological Q waves, (3) imaging evidence of new viable myocardium loss or regional wall motion abnormality consistent with ischemic origin, or (4) angiographic findings consistent with a procedural flow-limiting complication. Nevertheless, the post-PCI cutoff chosen to define type 4a MI after NSTEMI is consensus-based and lacks robust evidence (70).

A standardized, evidence-based definition of periprocedural ischemic events with prognostic relevance and clinical applicability is needed. Current literature mainly focuses on chronic coronary syndromes and elective CABG/PCI. However, NSTEMI patients often have complex clinical profiles with multiple comorbidities (age, diabetes, chronic kidney disease), making them more susceptible to ischemic events (72).

Studies on long-term prognostic implications of pMI, primarily after elective PCI (non-ACS), have yielded mixed results, with recent larger studies contradicting earlier findings of a significant association with 1-year major events and death (73). These mixed findings may stem from the difference in adopted biomarkers, highlighting the importance of careful pMI diagnostic criteria selection for prognostic outcomes.

In CABG, optimal thresholds for clinically significant periprocedural myocardial injury with prognostic significance remain debated. Current data suggest very high increments of ischemic biomarkers (both CK-MB and troponin) play a prognostic role, implying current pMI definitions may be excessively sensible (74).

Valuable data on pMI rates based on various “outdated” and contemporary definitions (SYNTAX, ISCHEMIA, EXCEL, SCAI, and Fourth UDMI) and their impact on 5-year cardiovascular mortality and 10-year all-cause mortality were derived from a long-term analysis of the SYNTAX trial.

Key differences among the definitions of pMI included the biomarker thresholds used to define MI and the requirement for additional supporting criteria. Definitions relying solely on biomarker elevations, even at high thresholds, returned higher MI rates than those requiring additional evidence following PCI or CABG, with this discrepancy being significantly more pronounced after CABG. After PCI, all definitions of PMI correlated with increased 5-year cardiovascular and 10-year all-cause mortality. In contrast, after CABG, only definitions with supporting criteria of ischemia criteria (ECG, imaging, angiography) showed a clear mortality increase (75).

An illustrative example about the controversy of the pMI definition is represented by the EXCEL trial (25). The European Association for Cardio-Thoracic Surgery (EACTS) withdrew its support for the 2018 ESC/EACTS guidelines on myocardial revascularization of LM-CAD because that guidelines assigned PCI a Class IIa recommendation for patients with intermediate SYNTAX scores, while CABG retained a Class Ia across all levels of anatomical complexity.

EACTS' withdrawal was based on concerns from the surgical community that data related to periprocedural MI in the PCI group had been misinterpreted. Specifically, the EXCEL investigators adopted a protocol-defined MI, which relied predominantly on creatine kinase–MB (CK-MB) elevations >10 times the upper reference limit, as the primary definition for pMI. This definition was chosen because, at the time, such elevations had been shown to impact prognosis and cardiovascular mortality. In contrast, the Third Universal Definition of MI (UDMI), which relied on more sensitive troponin measurements with a lower diagnostic threshold and additional ancillary criteria, was not accepted as protocol-definition for pMI.

Furthermore, the use of third UDMI was seen as potentially biased due to differing diagnostic criteria for PCI and CABG, and a lack of demonstrated correlation with outcomes. This position was reportedly agreed upon by all investigators involved in EXCEL, including surgical representatives, prior to the publication of the study protocol. Notably, the protocol remained unchanged throughout the study duration.

At the 3-year follow-up, the EXCEL trial reported no significant differences in the composite endpoint of death, MI, or stroke between patients with LM-CAD and low-to-intermediate SYNTAX scores treated with either PCI or CABG. Despite this, the EACTS contended that applying the UDMI to the full dataset would have resulted in an estimated 80% higher rate of pMI in the PCI arm compared to the CABG group. These data, although listed as a secondary endpoint in the original protocol, were not reported in the initial publication due to lack of troponin samples, which was optional to measure according to the study protocol.

In response to this controversy, a new substudy based on the EXCEL cohort was subsequently launched to assess pMI rates and clinical relevance using the Third Universal Definition of MI (UDMI) in EXCEL trial patients, despite including only around 13% of patients with NSTEMI. Due to the fact that the EXCEL protocol adopted CK-MB to define pMI, and troponin data were limited, UDMI was applied using CK-MB as a substitute or via a hybrid approach. Procedural MI, by EXCEL protocol, occurred more often after CABG than PCI. However, applying the Third UDMI (requiring supporting clinical criteria) substantially reduced procedural MI rates after CABG, while PCI rates remained similar. Importantly, protocol-defined procedural MI was associated with 5-year mortality after either CABG or PCI, but the UDMI definition was linked to mortality only after CABG (76).

SYNTAX and EXCEL investigators provided useful pMI data, but neither routinely included pre- or post-procedure troponin. In addition, differing and outdated pMI definitions and reliance on CK-MB over high-sensitivity troponin, limit current clinical applicability. In both trials, supporting ischemia criteria (ECG changes, imaging evidence, or documented vessel occlusion) significantly impacted MI diagnosis after CABG. Elevated biomarkers alone, even at high levels, increased procedural MI frequency but added little to its prognostic value for mortality. Conversely, for PCI patients, the threshold of high sensitivity biomarker seemed to be fundamental in diagnosing pMI.

SYNTAX and EXCEL findings support ancillary criteria for pMI after CABG. However, for PCI, these criteria offer limited value and should be omitted in favor of a standalone, prognostically significant biomarker's threshold. However, this supposed appropriate threshold remains debated.

A recent meta-analysis of PCI vs. CABG trials investigated pMI association with mortality and how definitions and ancillary criteria modified this. Despite heterogeneity, a consistent association between pMI and all-cause mortality was found when pMI was defined by substantial biomarker elevations (>5 times URL), also linked to increased cardiac mortality. However, lacking patient-level data limited confounding factor control. Trials with higher pMI incidence might have included higher baseline risk patients, potentially reflecting confounding rather than a direct causal link (77).

Recently, NSTEMI patients with stable or falling pre-PCI cTnI undergoing PCI were prospectively investigated for pMI and type 4a MI incidence and prognostic relevance per Fourth UDMI. This study revealed that around 40% of NSTEMI developed pMI and this is associated with higher 1-year all-cause mortality and major ischemic events. Those with pMI meeting type 4a MI criteria faced significantly elevated adverse clinical outcomes, including mortality.

Type 4a MI's defining characteristic is clinical and diagnostic features of myocardial ischemia directly related to revascularization procedure. Thus, identifying new ischemic ECG/echo changes or angiographic evidence of a flow-limiting complication, offers prognostic value beyond post-PCI troponin elevation alone (78).

The study also addressed the prognostically meaningful troponin elevation threshold after NSTEMI revascularization. A post-PCI troponin elevation between 20% and 40% showed similar 1-year outcomes as increases below 20%. In contrast, an increase superior to 40% combined with an absolute post-procedural value ≥5 times the 99th URL was optimal for diagnosing prognostically significant pMI. Patients exceeding this threshold faced a fourfold increase in 1-year all-cause mortality and a threefold increase in strong cardiovascular events (78).

NSTEMI patients have a remarkably higher incidence of type 4a MI than chronic coronary syndromes, suggesting acute setting factors contribute to the increased risk (71). The acute NSTEMI phase involves active inflammation, plaque instability, and endothelial dysfunction, favoring a prothrombotic state (79). During PCI, vulnerable plaque disruption and mechanical injury can trigger angiographically evident thrombus formation, leading to type 4a MI (80). During PCI, these high-risk lesions are more often associated with distal embolization, contributing to microvascular obstruction and myocardial injury. The acute inflammatory environment in NSTEMI, with cytokine/chemokine release, may increase myocardial injury susceptibility by promoting endothelial dysfunction, increasing microvascular resistance, and impairing myocardial perfusion, increasing no-reflow risk during PCI (81).

The inflammatory burden in NSTEMI can worsen myocardial injury during CABG, particularly when surgery occurs during the acute phase soon after symptom onset. Although data in the context of ACS are limited, intraoperative hypoperfusion due to technical factors such as cardiac manipulation, clamping for cardiopulmonary bypass, cardioplegic arrest, ischemia-reperfusion injury, or elevated left ventricular diastolic pressure, likely contributes to pMI. However, these factors appear to have limited impact on long-term prognosis. In contrast, graft failure due to thrombosis or restenosis meaningfully affects long-term outcomes and is often accompanied by both elevated troponin levels and objective evidence of ischemia. Therefore, post-CABG assessment should not rely solely on troponin measurements but should incorporate additional markers of ischemia whenever possible.

Given the significant prognostic implications of pMI after NSTEMI, there is a need for precise, individualized risk stratification and targeted therapeutic strategies to improve patient outcomes. Still, it remains unclear whether the observed increases in mortality and cardiovascular events are primarily driven by procedural complexity, patient vulnerability or iatrogenic myocardial injury. Further research is essential to better define the true prognostic significance of pMI and to determine whether its definition and diagnostic criteria should be tailored to the mode of revascularization. If future evidence fails to demonstrate a strong, independent association between pMI and adverse clinical outcomes, its inclusion as a component of primary composite endpoints in clinical trials may warrant reevaluation.

## Pretreatment with P2Y12 inhibitor in NSTEMI

Pretreatment with an oral P2Y12 receptor inhibitor, in addition to aspirin, refers to its administration to all patients prior to coronary angiography—regardless of whether the diagnosis of NSTEMI is ultimately confirmed and whether PCI is subsequently indicated. In randomized clinical trials, only about 65% of carefully selected patients undergo both confirmation of the diagnosis and subsequent PCI (82), and this percentage is even lower in real-world registries (83).

For these 65% of patients, pretreatment is intuitively appealing, as early administration allows sufficient time for oral antiplatelet agents to reach optimal efficacy by the time of PCI. This could potentially enhance protection against thrombotic complications related to the procedure, preventing early stent thrombosis, and decreasing the need for glycoprotein IIb/IIIa inhibitor bailout us. However, the remaining 35% of patients referred to the cath-lab may be exposed to unnecessary bleeding risks. Moreover, if emergent CABG is required, these patients may face an increased risk of perioperative bleeding.

These risks have sparked significant debate over the optimal timing of P2Y12 receptor inhibitor administration in ACS, particularly in NSTEMI.

Importantly, the latest edition of the ESC Guidelines for the management of NSTE-ACS downgraded routine pretreatment strategy with P2Y12 receptor inhibitors to non-recommended in the acute setting (1).

The study most often cited by opponents of pretreatment is the previously mentioned ISAR-REACT 5 trials (84). Approximately 46% of the trial participants presented with NSTEMI. In this study, patients randomized to the ticagrelor arm received a loading dose as early as possible after randomization. In contrast, patients assigned to prasugrel received the loading dose only after coronary anatomy had been assessed and PCI was deemed necessary.

At one-year post-randomization, a composite of death from any cause, MI or stroke occurred significantly more frequently in the ticagrelor group compared to the prasugrel group. Regarding safety, there was no statistically significant difference between the two groups in the incidence of Bleeding Academic Research Consortium (BARC) type 3, 4, or 5 bleeding events. The benefit in the prasugrel arm was primarily due to a 1.8 percentage point reduction in the rate of recurrent MI, both spontaneous and peri-procedural.

Notably, a benefit of prasugrel was not demonstrated in the trial in patients with ACS managed conservatively.

Nevertheless, the use of an intention-to-treat analysis had a significant impact on the results, as over 20% of patients were discharged on a different antiplatelet agent than the one they were randomized to receive. In total, 1,299 patients included in the final analysis were not treated with their originally assigned drug. Considering these methodological issues, along with the exclusion of a high number of participants from the final analysis, make it difficult to view the ISAR-REACT 5 results as definitive or ground-breaking. Despite this, the trial is among those cited by the ESC Guidelines in shifting the paradigm on pretreatment, leading to its downgrading to a class III recommendation.

The ACCOAST trial (85) was the most important, well-designed and comprehensive randomized study focused on evaluating the pretreatment strategy in NSTEMI patients. It enrolled 4,033 patients with NSTEMI, who were randomly assigned or to a pretreatment arm that received 30 mg of prasugrel before angiography and another 30 mg if PCI was needed, and a control arm that received placebo before angiography and 60 mg of prasugrel only in case of PCI. If CABG was required, the pretreatment group did not receive the second dose, and the control group did not receive prasugrel at all (86).

PCI was ultimately performed in 68.7% of patients, with a median time of 4.3 h after the loading dose. Within 7 days, 6.2% of patients required CABG and 25.1% was medically managed.

The primary composite endpoint including cardiovascular death, MI, stroke, urgent revascularization or GP IIb/IIIa inhibitor rescue therapy, did not differ significantly between the two arms. In addition, no significant differences were observed in any individual component of the primary endpoint and in total mortality at day 7 or 30.

Importantly, pretreatment did not lower neither ischemic events during the waiting period for angiography, (0.8% in the pretreatment arm vs. 0.9% in the control arm) nor stent thrombosis rates at 30-days (0.1% in the pretreatment arm and 0.4% in the control arm).

In addition, a pharmacodynamic substudy showed greater platelet inhibition in the pretreatment group at the time of arterial access (median 4.8 h after the initial dose), likely contributing to the increased bleeding risk. Two hours after the second loading dose, both groups had similarly low platelet reactivity, sustained for up to 24 h.

In terms of safety, the rate of all Thrombolysis in Myocardial Infarction (TIMI) major bleeding episodes, including both CABG-related bleedings and non-CABG, was meaningfully increased with pretreatment. The rates of TIMI major bleeding and life-threatening bleeding not related to CABG were increased by a factor of 3 and 6, respectively. Notwithstanding there was no significant difference between groups in CABG-related bleeding alone, despite the pretreatment arm showed an increasing trend.

Randomized data carefully assessing the impact of a pretreatment strategy with potent P2Y12 inhibitors remain limited and inconclusive.

The DUBIUS (87) aimed to discover any significant difference between either upstream treatment with ticagrelor or a downstream strategy (prasugrel or ticagrelor) in candidates to PCI. The trial was terminated early due to the unexpectedly low incidence of both ischemic and bleeding events. Notwithstanding, at 30 days, there was no significant difference in major events the upstream and downstream groups. Likewise, bleeding events classified as BARC types 3, 4, and 5 occurred at similar rates in both groups.

In addressing the question of ischemic risk associated with deferring P2Y12 inhibitor pretreatment in NSTEMI, it's important to note that the time from admission to coronary angiography has significantly decreased days (88) to hours (86) over the years.

The risk of clinical deterioration, such as dynamic ST-segment changes or hemodynamic instability, during the PCI-waiting period is low. A subgroup analysis from the ACCOAST (89), evaluated the impact of pretreatment on early adverse events and found that pretreatment did not prevent major ischemic event, regardless of the timing of angiography within the first 48 h.

Similarly, the DUBIUS (87) supported these findings, showing comparable event rates regardless of pretreatment status for patients undergoing angiography either within 24 h or between 24 and 72 h.

Given this clear evidence against routine upstream administration of oral P2Y12 inhibitors, the latest ESC Guidelines (1) for the management of NSTE-ACS appeared to favor alternatives such as cangrelor, in NSTEMI at high ischemic risk, despite data supporting its use are not yet definitive.

Cangrelor favorable characteristics include a rapid onset/offset of action and a solid dose-dependent antiplatelet effect. It has been evaluated in the setting of botch chronic and acute setting in the CHAMPION trials.

CHAMPION PCI (90) (49% NSTEMI) and CHAMPION PLATFORM (91) (60% NSTEMI) failed to demonstrate a reduction in the composite endpoint of death, myocardial infarction, or ischemia-driven revascularization at 48 h with cangrelor compared to placebo or clopidogrel. In contrast, CHAMPION PHOENIX (92) (25% NSTEMI) showed that cangrelor significantly lowered ischemic events during PCI without increasing major bleeding. Specifically, the observed reduction in the ischemic endpoint was driven mainly by lower rates of stent thrombosis and periprocedural MI.

A patient-level meta-analysis of the CHAMPION trials (93), which included 57% NSTEMI patients, confirmed a significant reduction in periprocedural events, such as stent thrombosis, periprocedural MI and GP IIb/IIIa inhibitors bailout. Further, in terms of safety, while minor bleeding increased, rates of life-threatening or major bleeding were comparable between groups.

These findings have supported the guideline-endorsed use of cangrelor in ACS patients who are P2Y12 inhibitor–naïve, especially in high ischemic risk, to enhance platelet inhibition during PCI. This mainly because oral P2Y12 inhibitors, despite their potency, have a slower onset and their absorption might be unpredictable, especially in acute settings.

In real-world high-risk populations, cangrelor offers the advantage of rapid platelet inhibition and quick recovery after discontinuation. This rapid reversibility makes cangrelor a valuable option when urgent surgery is needed. This is proven in the BRIDGE study (76), where cangrelor demonstrated significantly greater platelet inhibition without excess bleeding among patients awaiting cardiac surgery.

In exploring the issue of P2Y12 inhibitor administration prior to CABG, more data may be extrapolated from outdated studies and from real world registries. In the CURE trial, bleeding complications were notably more frequent in patients undergoing CABG, particularly when clopidogrel was discontinued less than 5 days before surgery. As a result, guidelines recommend delaying CABG for 5–7 days after stopping clopidogrel (88).

This association has been supported by other studies. In the CRUSADE registry (94), patients who underwent CABG within 5 days of discontinuing clopidogrel had a significant increase in blood transfusions. However, if surgery was delayed for at least 5 days, bleeding risks were similar between clopidogrel-treated patients and non-clopidogrel-treated.

Additionally, a multicenter retrospective cohort study highlighted that NSTEMI requiring CABG non-discontinuing clopidogrel in the 5 days before surgery, showed higher rates of reoperation, major bleeding and prolonged hospital stays (95).

A more recent study, the ACUITY trial (82), focused on patients with NSTE-ACS undergoing early invasive management and provided additional insight into clopidogrel use before CABG. Among the 11% of patients who underwent CABG before discharge, clopidogrel-treated ones had a longer median hospital stay but experienced composite ischemic events at 30 days. Importantly, rates of non-CABG-related and post-CABG major bleeding were not significantly different compared to non-clopidogrel-treated patients. Multivariable analysis confirmed that clopidogrel use before CABG was independently associated with a reduction in 30-day ischemic events, mainly driven by fewer myocardial infarctions, without increasing major bleeding.

The timing of surgery relative to clopidogrel exposure played a key role. Patients who received clopidogrel and had surgery ≥5 days after the last dose experienced significantly lower rates of both 30-day and 1-year ischemic events and required fewer transfusions than those unexposed.

When combining ischemic and bleeding outcomes into a net adverse clinical event analysis, early clopidogrel administration followed by a delay of ≥5 days before CABG resulted in the most favorable strategy.

The idea of avoiding pretreatment In NSTEMI in everyday clinical practice to avoid postponing surgery, reducing major bleeding and hospital length of stay emerges both from randomized data and from real-world data. Another important topic in NSTEMI in the everyday clinical practice is the misdiagnosis.

The approach of avoiding pretreatment in NSTEMI patients in routine clinical practice stems from both randomized and real-world data, aiming to reduce surgical delays, major bleeding and hospital stay. However, another key challenge in everyday management is the risk of misdiagnosis.

A significant proportion of patients are initially misdiagnosed with NSTEMI. Even in carefully designed studies, diagnostic inaccuracies are remarkable. For example, in the ACUITY (82), around 30% of patients enrolled were later found not to have coronary stenosis.

As a result, early initiation of P2Y12 inhibitors can delay necessary surgical revascularization procedures. The ACCOAST (85) supported a more targeted approach, showing that deferring prasugrel until after angiography is both safe and effective. This strategy ensures that P2Y12 inhibitors are given only to those undergoing PCI, sparing patients who require CABG from unnecessary treatment delays.

The Swedish Coronary Angiography and Angioplasty Registry (SCAAR), involving more than 64000 patients who underwent PCI for NSTE-ACS between 2010 and 2018, showed that a pretreatment strategy was not associated with improved survival or a reduced incidence of definite stent thrombosis. On the contrary, it was linked to an increased risk of bleeding. A shift in clinical practice from routine pretreatment to no pretreatment corresponded with a significant reduction in bleeding risk. This was proven by a prospective evaluation, which was part of the study, were, following the change clinical practice, the use of pretreatment declined from 99% in 2010 to just 15% in 2018. This shift did not reveal any significant difference in short and long-term mortality nor ischemic events. Indeed, when routine pretreatment was largely discontinued, the risk of bleeding drastically dropped. Patients who underwent CABG, during the non-pretreatment period, showed a reduced rate of major bleeding and a reduced rate of reoperation due to bleeding (83).

In conclusion, the use of pretreatment with P2Y12 inhibitors has significantly declined in recent years, although some non-tertiary centers continue to follow this strategy despite the growing body of evidence against it.

For patients admitted with a working diagnosis of NSTEMI, initial management should include aspirin and anticoagulation, alongside planning for coronary angiography. When immediate angiography is not possible due to the need for patient transfer, the procedure should be performed within 48–72 h. In such cases, a pretreatment strategy may be considered.

However, a routine pretreatment approach is not only unnecessary but may be harmful, particularly in patients at high risk of bleeding or in younger individuals who may ultimately require surgical revascularization. The optimal time for P2Y12 inhibitor administration is only when coronary anatomy is defined and a decision for PCI is made.

For patients undergoing high-risk PCI, cangrelor represents a promising option to achieve immediate and potent platelet inhibition. This may be particularly useful in scenarios where PCI of the culprit lesion is performed, but subsequent complete surgical revascularization is. While evidence in this setting is still limited, a potential future role for cangrelor may arise in two common clinical scenarios. The first in NSTEMI patients who have not received a loading dose of a potent P2Y12 inhibitor and require bridging to surgery during hospitalization, whether due to diffuse coronary disease or anatomy unsuitable for PCI. The second, in NSTEMI patients who have already received a loading dose for culprit-lesion PCI and require bridging to CABG, where cangrelor may help reduce both the risk of stent thrombosis while awaiting surgery and bleeding during the surgery when stopped before.

In all these cases, close collaboration between interventional cardiologists and cardiac surgeons is crucial, especially for patients who may benefit most from surgical revascularization, such as younger individuals and those with complex coronary anatomy.

## Timing of surgery: when late is too late?

Once CABG is chosen as the revascularization method, the next consideration is the timing of the surgery. A large study of pooled data from several North American ACS-databases compared in-hospital outcomes in this setting. Among more than 100,000 patients with NSTEMI, 2,647 underwent CABG during their index admission, with half of them who underwent surgery more than 48 h after admission. The study found no significant differences in unadjusted in-hospital outcomes such as death, postoperative MI, congestive heart failure, shock, or stroke between early and late CABG groups and these findings held after multivariable adjustment. Interestingly, patients undergoing CABG after 48 h were more likely to present with heart failure and had a higher surgical risk. The authors concluded that CABG timing is influenced significantly by upstream decisions regarding initial strategy and medical management, stressing its role in the therapeutic pathway (34).

A German prospective study found no difference in mortality and major cardiovascular events at 30-days and 6-months between patients who underwent early or late CABG (within or after 72 h from symptom onset) following NSTE-ACS. The early-CABG group had more patients in cardiogenic shock and higher incidences of mechanical circulatory support use. Intraoperative variables such as pump and cross-clamp times and the number of anastomoses were similar between groups, but the early-CABG group had a significantly higher rate of incomplete revascularization. It is unclear whether this was due to the severity of CAD or the overall condition of the patients (96).

A more recent cohort study examined outcomes based on the timing of CABG (within 24 h or >24 h from presentation). As expected, the group undergoing CABG within 24 h was smaller and had more severe illness, with higher rates of failed PCI and cardiogenic shock. Unadjusted operative mortality was higher in early-CABG group; however, after risk adjustment and propensity matching, there was no significant difference in operative mortality between the two-timing groups (97).

On the other hand, a large U.S. registry enrolling 40,000 patients hospitalized for acute MI (45% of NSTEMI) undergoing CABG, showed that the early-CABG (within the first two days) had higher mortality rates compared to those who received surgery three or more days after the acute event. These findings suggest that, in non-emergent scenarios, deferring CABG until at least three days after admission for ACS may be associated with improved outcomes (98).

In support for these results, data from the National Inpatient Sample in the US (2009–2018) analyzing outcomes for NSTEMI patients undergoing CABG, grouped by the interval days-to-surgery (0, 1–3, 4–7 and >7 days), revealed that revascularization performed on days 1–3 and 4–7 resulted in comparable in-hospital mortality rates. Contrarywise, mortality was higher for procedures done on day 0 (too-early CABG) and after day 7 (too-late CABG). This may be explained by the fact that acute surgical revascularization, performed at the peak of myocardial inflammatory response, involving cardiac manipulation, cardioplegic arrest and cardiopulmonary bypass, may exacerbate microcirculatory hypoperfusion, increasing ischemic injury, potentially diminishing the benefits of revascularization. At the same time, it remains difficult to explain why patients undergoing surgical revascularization beyond day 7 do not appear to retain the prognostic advantage observed in other subgroups. However, these results support the recommendation to perform revascularization within 1–3 days when clinically appropriate and feasible (99).

Formulating a definitive recommendation for the optimal timing of CABG after ACS is challenging. However, data suggest that the highest risk period is within the first 24 h, often associated with salvage scenarios. The appropriate interval between ACS and CABG largely depends on a patient's comorbidities, clinical presentation and prior treatments. In this context as well, interdisciplinary discussion is essential, with the Heart Team playing a pivotal role in guiding the decision-making process.

## On-pump and Off-pump CABG in ACS

The best surgical strategy for CABG remains debated, particularly with the emerging alternatives to conventional cardiopulmonary bypass and cardioplegic arrest. Off-pump CABG is believed to offer myocardial protection by preserving coronary flow, avoiding reperfusion injury, enabling earlier revascularization and reducing myocardial oedema. It also avoids the adverse effects of cardioplegic arrest, and, as well, extensive aortic manipulation, hemodilution and hypothermia.

On-pump beating heart CABG may reduce surgical time, avoid the hemodynamic effects of cardiac manipulation associated with off-pump CABG. However, beating heart surgery is technically demanding, raising concerns about the completeness of revascularization and long-term graft patency achieved off-pump (100).

In the setting of ACS, older data suggested that beating heart surgery may not be feasible or tolerated in hemodynamically compromised patients. A review of surgical outcomes showed that off-pump CABG was the predominant strategy, with the proportion of on-pump beating CABG increasing in higher-risk subgroups. Average observed mortality rates aligned with preoperative estimated risk across all subgroups, but off-pump resulted in significantly lower mortality and major complications in the patient at non-high surgical risk (101).

A meta-analysis of several RCTs and observational studies analyzing 30-day mortality and a composite of cardiovascular strong events in ACS patients undergoing on-pump, off-pump, and on-pump beating heart CABG showed significant difference between these surgical approaches. However, the mortality benefit with off-pump in AMI patients suggests that high-acuity patients may benefit most from avoiding the myocardial injury associated with cardiopulmonary bypass and cardioplegic arrest (102).

Cardiac surgeons are often discouraged from converting off-pump to on-pump CABG because it typically results in worse post-operative outcomes, a higher rate of complications, and increased in-hospital mortality (103–105). However, with continuous experience and skill, both early and late outcomes of off-pump are similar to on-pump, as demonstrated by results from dedicated high-volume centers (106).

A recent meta-analysis involving 3,001 patients (817 off-pump, 2,184 on-pump CABG) found that off-pump had comparable mortality to on-pump CABG at both 30 days and mid-term follow-up. Off-pump was associated with less complete revascularization and a lower revascularization index, though there was no difference in re-intervention rates. New emphasis should be placed on off-pump CABG, considering as a safe and comparable alternative to on-pump CABG for clinically stable ACS patients requiring revascularization. However, further research is needed to define selection criteria, better characterize this heterogeneous patient group and assess the effects of incomplete revascularization on long-term outcomes (107).

## Conclusion

The ongoing debate over the optimal revascularization strategy in myocardial infarction with non-ST-segment elevation emphasize the complexity of balancing clinical outcomes with procedural risks.

Despite the available evidence in long-term outcomes provided by CABG, particularly in terms of MI and repeat revascularization, especially in patients with anatomically complex disease or comorbidities, PCI remains less invasive, more widely available and with shorter recovery time, making it appealing in emergent situations or patients at high surgical risk. Notwithstanding, PCI carries often the risk of incomplete revascularization and high rates of reintervention.

In the context of NSTEMI, due to patients comorbities, anatomical complexity, age and surgical risk the choice between PCI and CABG demands a tailored, patient-centered approach. On this purpose the Heart Team plays a pivotal role in guiding the decision-making process, ensuring an individualized treatment strategy.

Despite substantial progress, gaps in the literature remain. Future research should focus on filling the gaps widely discussed in this review through robust clinical trials, assessing clinical outcomes over extended follow-up periods and evaluating the impact of newer generation materials and novel percutaneous and surcial techniques. Until more definitive high-quality evidence becomes available, the integration of clinical guidelines and a multidisciplinary evaluation will play a central role in optimizing the revascularization strategy in NSTEMI. This multidisciplinary approach ensures that the therapeutic decisions are tailored to every specific patient in line with the evolving evidence.

## References

[1] Byrne RA Rossello X Coughlan JJ Barbato E Berry C Chieffo A 2023 ESC guidelines for the management of acute coronary syndromes. Eur Heart J. (2023) 44(38):3720–826. 10.1093/eurheartj/ehad191 37622654

[2] Rao SV O’Donoghue ML Ruel M Rab T Tamis-Holland JE Alexander JH 2025 ACC/AHA/ACEP/NAEMSP/SCAI guideline for the management of patients with acute coronary syndromes: a report of the American college of cardiology/American heart association joint committee on clinical practice guidelines. Circulation. (2025) 151(13):e771–862. 10.1161/CIR.0000000000001328 40014670

[3] Omerovic E Råmunddal T Petursson P Angerås O Rawshani A Jha S Percutaneous vs. surgical revascularization of non-ST-segment elevation myocardial infarction with multivessel disease: the SWEDEHEART registry. Eur Heart J. (2025) 46(6):518–31. 10.1093/eurheartj/ehae700 39601339 PMC11804248

[4] Sipahi I Akay MH Dagdelen S Blitz A Alhan C. Coronary artery bypass grafting vs percutaneous coronary intervention and long-term mortality and morbidity in multivessel disease : meta-analysis of randomized clinical trials of the arterial grafting and stenting ERA. JAMA Intern Med. (2014) 174(2):223–30. 10.1001/jamainternmed.2013.12844 24296767

[5] Park S-J Ahn J-M Kim Y-H Park D-W Yun S-C Lee J-Y Trial of everolimus-eluting stents or bypass surgery for coronary disease. N Engl J Med. (2015) 372(13):1204–12. 10.1056/NEJMoa1415447 25774645

[6] Park S-J Kim Y-H Park D-W Yun S-C Ahn J-M Song HG Randomized trial of stents versus bypass surgery for left main coronary artery disease. N Engl J Med. (2011) 364(18):1718–27. 10.1056/NEJMoa1100452 21463149

[7] Serruys PW Morice M-C Kappetein AP Colombo A Holmes DR Mack MJ Percutaneous coronary intervention versus coronary-artery bypass grafting for severe coronary artery disease. N Engl J Med. (2009) 360(10):961–72. 10.1056/NEJMoa0804626 19228612

[8] Chang M Lee CW Ahn J-M Cavalcante R Sotomi Y Onuma Y Comparison of outcome of coronary artery bypass grafting versus drug-eluting stent implantation for non–ST-elevation acute coronary syndrome. Am J Cardiol. (2017) 120(3):380–6. 10.1016/j.amjcard.2017.04.038 28595861

[9] Klempfner R Barac YD Younis A Kopel E Younis A Ronen G Early referral to coronary artery bypass grafting following acute coronary syndrome, trends and outcomes from the acute coronary syndrome Israeli survey (ACSIS) 2000–2010. Heart Lung Circ. (2018) 27(2):175–82. 10.1016/j.hlc.2017.01.017 28325709

[10] Shiyovich A Shlomo N Cohen T Iakobishvili Z Kornowski R Eisen A. Temporal trends of patients with acute coronary syndrome and multi-vessel coronary artery disease—from the ACSIS registry. Int J Cardiol. (2020) 304:8–13. 10.1016/j.ijcard.2020.01.040 32033784

[11] Ram E Sternik L Klempfner R Iakobishvili Z Peled Y Shlomo N Outcomes of different revascularization strategies among patients presenting with acute coronary syndromes without ST elevation. J Thorac Cardiovasc Surg. (2020) 160(4):926–935.e6. 10.1016/j.jtcvs.2019.08.130 31653430

[12] Huckaby LV Sultan I Mulukutla S Kliner D Gleason TG Wang Y Revascularization following non-ST elevation myocardial infarction in multivessel coronary disease. J Card Surg. (2020) 35(6):1195–201. 10.1111/jocs.14539 32362025 PMC9057453

[13] Mehaffey JH Hayanga JWA Kawsara M Sakhuja A Mascio C Rankin JS Contemporary coronary artery bypass grafting vs multivessel percutaneous coronary intervention. Ann Thorac Surg. (2023) 116(6):1213–20. 10.1016/j.athoracsur.2023.05.032 37353103 PMC10739562

[14] Kakar H Groenland FTW Elscot JJ Rinaldi R Scoccia A Kardys I Percutaneous coronary intervention versus coronary artery bypass grafting in non–ST-elevation coronary syndromes and multivessel disease: a systematic review and meta-analysis. Am J Cardiol. (2023) 195:70–6. 10.1016/j.amjcard.2023.03.005 37011556

[15] Jia S Zhang C Jiang L Xu L Tian J Zhao X Comparison of percutaneous coronary intervention, coronary artery bypass grafting and medical therapy in non-ST elevation acute coronary syndrome patients with 3-vessel disease. Circ J. (2020) 84(10):1718–27. 10.1253/circj.CJ-20-0300 32848116

[16] Liakopoulos OJ Slottosch I Wendt D Welp H Schiller W Martens S Surgical revascularization for acute coronary syndromes: a report from the North Rhine-Westphalia surgical myocardial infarction registry. Eur J Cardiothorac Surg. (2020) 58(6):1137–44. 10.1093/ejcts/ezaa260 33011789

[17] Fearon WF Zimmermann FM Ding VY Takahashi K Piroth Z van Straten AHM Outcomes after fractional flow reserve-guided percutaneous coronary intervention versus coronary artery bypass grafting (FAME 3): 5-year follow-up of a multicentre, open-label, randomised trial. Lancet. (2025) 405(10488):1481–90. 10.1016/S0140-6736(25)00505-7 40174598

[18] Sabik JF Bakaeen FG Ruel M Moon MR Malaisrie SC Calhoon JH The American association for thoracic surgery and the society of thoracic surgeons reasoning for not endorsing the 2021 ACC/AHA/SCAI coronary revascularization guidelines. Ann Thorac Surg. (2022) 113(4):1065–8. 10.1016/j.athoracsur.2021.12.003 34954249

[19] Myers PO Beyersdorf F Sadaba R Milojevic M. European association for cardio-thoracic surgery statement regarding the 2021 American heart association/American college of cardiology/society for cardiovascular angiography and interventions coronary artery revascularization guidelines. Eur J Cardiothorac Surg. (2022) 62(1):ezac060. 10.1093/ejcts/ezac060 35349675

[20] Soca G Martínez A Gomes WJ Solano JG Almeida R Ibañez MA The South American society of cardiology (SSC) and the Latin American association of cardiac and endovascular surgery (LACES) statement on the 2021 ACC/AHA/SCAI guidelines for coronary artery revascularization. Braz J Cardiovasc Surg. (2023) 38(5):e20230119. 10.21470/1678-9741-2023-0119 37796841 PMC10548594

[21] Desperak P Hawranek M Gąsior P Desperak A Lekston A Gąsior M. Long-term outcomes of patients with multivessel coronary artery disease presenting non-st-segment elevation acute coronary syndromes. Cardiol J. (2019) 26(2):157–68. 28980282 10.5603/CJ.a2017.0110 PMC8086658

[22] Widmer RJ Hammonds K Mixon T Exaire JE Chiles CD Tavilla G Acute coronary syndrome revascularization strategies with multivessel coronary artery disease. Am J Cardiol. (2024) 220:33–8. 10.1016/j.amjcard.2024.04.003 38582315

[23] Palmerini T Serruys P Kappetein AP Genereux P Riva DD Reggiani LB Clinical outcomes with percutaneous coronary revascularization vs coronary artery bypass grafting surgery in patients with unprotected left main coronary artery disease: a meta-analysis of 6 randomized trials and 4,686 patients. Am Heart J. (2017) 190:54–63. 10.1016/j.ahj.2017.05.005 28760214

[24] Thuijs DJFM Kappetein AP Serruys PW Mohr F-W Morice M-C Mack MJ Percutaneous coronary intervention versus coronary artery bypass grafting in patients with three-vessel or left main coronary artery disease: 10-year follow-up of the multicentre randomised controlled SYNTAX trial. Lancet. (2019) 394(10206):1325–34. 10.1016/S0140-6736(19)31997-X 31488373

[25] Stone GW Kappetein AP Sabik JF Pocock SJ Morice M-C Puskas J Five-year outcomes after PCI or CABG for left main coronary disease. N Engl J Med. (2019) 381(19):1820–30. 10.1056/NEJMoa1909406 31562798

[26] Park D-W Ahn J-M Park H Yun S-C Kang D-Y Lee PH Ten-year outcomes after drug-eluting stents versus coronary artery bypass grafting for left main coronary disease: extended follow-up of the PRECOMBAT trial. Circulation. (2020) 141(18):1437–46. 10.1161/CIRCULATIONAHA.120.046039 32223567

[27] Holm NR Mäkikallio T Lindsay MM Spence MS Erglis A Menown IBA Percutaneous coronary angioplasty versus coronary artery bypass grafting in the treatment of unprotected left main stenosis: updated 5-year outcomes from the randomised, non-inferiority NOBLE trial. Lancet. (2020) 395(10219):191–9. 10.1016/S0140-6736(19)32972-1 31879028

[28] Gaba P Christiansen EH Nielsen PH Murphy SA O’Gara PT Smith PK Percutaneous coronary intervention vs coronary artery bypass graft surgery for left main disease in patients with and without acute coronary syndromes: a pooled analysis of 4 randomized clinical trials. JAMA Cardiol. (2023) 8(7):631–9. 10.1001/jamacardio.2023.1177 37256598 PMC10233454

[29] Vergallo R Crea F. Atherosclerotic plaque healing. N Engl J Med. (2020) 383(9):846–57. 10.1056/NEJMra2000317 32846063

[30] Kirov H Caldonazo T Rahouma M Robinson NB Demetres M Serruys PW A systematic review and meta-analysis of percutaneous coronary intervention compared to coronary artery bypass grafting in non-ST-elevation acute coronary syndrome. Sci Rep. (2022) 12(1):5138. 10.1038/s41598-022-09158-0 35332253 PMC8948200

[31] Moussa ID Mohananey D Saucedo J Stone GW Yeh RW Kennedy KF Trends and outcomes of restenosis after coronary stent implantation in the United States. J Am Coll Cardiol. (2020) 76(13):1521–31. 10.1016/j.jacc.2020.08.002 32972528

[32] Scirica BM Bergmark BA Morrow DA Antman EM Bonaca MP Murphy SA Nonculprit lesion myocardial infarction following percutaneous coronary intervention in patients with acute coronary syndrome. J Am Coll Cardiol. (2020) 75(10):1095–106. 10.1016/j.jacc.2019.12.067 32164882

[33] Daoulah A Alqahtani AH Elmahrouk A Yousif N Almahmeed W Arafat AA Percutaneous coronary intervention vs. coronary artery bypass grafting in emergency and non-emergency unprotected left-main revascularization. Eur J Med Res. (2023) 28(1):210. 10.1186/s40001-023-01189-1 37393361 PMC10314560

[34] Thielmann M Wendt D Slottosch I Welp H Schiller W Tsagakis K Coronary artery bypass graft surgery in patients with acute coronary syndromes after primary percutaneous coronary intervention: a current report from the north-rhine westphalia surgical myocardial infarction registry. J Am Heart Assoc. (2021) 10(18):e021182. 10.1161/JAHA.121.021182 34514809 PMC8649544

[35] Bagheri J Jameie M Saryazdi ZD Jalali A Rezaee M Pashang M Coronary artery bypass graft surgery after primary percutaneous coronary intervention in patients with ST-elevation myocardial infarction. Heart Lung Circ. (2023) 32(10):1257–68. 10.1016/j.hlc.2023.08.005 37741752

[36] Massoudy P Thielmann M Lehmann N Marr A Kleikamp G Maleszka A Impact of prior percutaneous coronary intervention on the outcome of coronary artery bypass surgery: a multicenter analysis. J Thorac Cardiovasc Surg. (2009) 137(4):840–5. 10.1016/j.jtcvs.2008.09.005 19327506

[37] Thielmann M Neuhäuser M Knipp S Kottenberg-Assenmacher E Marr A Pizanis N Prognostic impact of previous percutaneous coronary intervention in patients with diabetes mellitus and triple-vessel disease undergoing coronary artery bypass surgery. J Thorac Cardiovasc Surg. (2007) 134(2):470–6. 10.1016/j.jtcvs.2007.04.019 17662792

[38] Thielmann M Leyh R Massoudy P Neuhäuser M Aleksic I Kamler M Prognostic significance of multiple previous percutaneous coronary interventions in patients undergoing elective coronary artery bypass surgery. Circulation. (2006) 114(SUPPL. 1):I-441. 10.1161/CIRCULATIONAHA.105.001024 16820616

[39] Ramanathan K Abel JG Park JE Fung A Mathew V Taylor CM Surgical versus percutaneous coronary revascularization in patients with diabetes and acute coronary syndromes. J Am Coll Cardiol. (2017) 70(24):2995–3006. 29241487 10.1016/j.jacc.2017.10.029

[40] Farkouh ME Domanski M Dangas GD Godoy LC Mack MJ Siami FS Long-term survival following multivessel revascularization in patients with diabetes: the FREEDOM follow-on study. J Am Coll Cardiol. (2019) 73(6):629–38. 10.1016/j.jacc.2018.11.001 30428398 PMC6839829

[41] Ben-Gal Y Mohr R Feit F Ohman EM Kirtane A Xu K Surgical versus percutaneous coronary revascularization for multivessel disease in diabetic patients with non-ST-segment-elevation acute coronary syndrome: analysis from the acute catheterization and early intervention triage strategy trial. Circ Cardiovasc Interv. (2015) 8(6):e002032. 10.1161/CIRCINTERVENTIONS.114.002032 26019142

[42] Bangalore S Guo Y Samadashvili Z Blecker S Xu J Hannan EL. Everolimus eluting stents versus coronary artery bypass graft surgery for patients with diabetes mellitus and multivessel disease. Circ Cardiovasc Interv. (2015) 8(7):e002626. 10.1161/CIRCINTERVENTIONS.115.002626 26156152 PMC6330115

[43] Kaul U Bangalore S Seth A Arambam P Abhaichand RK Patel TM Paclitaxel-Eluting versus everolimus-eluting coronary stents in diabetes. N Engl J Med. (2015) 373(18):1709–19. 10.1056/NEJMoa1510188 26466202

[44] Harskamp RE Bonatti JO Zhao DX Puskas JD de Winter RJ Alexander JH Standardizing definitions for hybrid coronary revascularization. J Thorac Cardiovasc Surg. (2014) 147(2):556–60. 10.1016/j.jtcvs.2013.10.019 24280716

[45] Tatoulis J Buxton BF Fuller JA. Patencies of 2,127 arterial to coronary conduits over 15 years. Ann Thorac Surg. (2004) 77(1):93–101. 10.1016/S0003-4975(03)01331-6 14726042

[46] BARI Investigators. The final 10-year follow-up results from the BARI randomized trial. J Am Coll Cardiol. (2007) 49(15):1600–6. 10.1016/j.jacc.2006.11.048 17433949

[47] Bonatti J Lehr E Vesely MR Friedrich G Bonaros N Zimrin D. Hybrid coronary revascularization: which patients? When? How? Curr Opin Cardiol. (2010) 25(6):568–74. 10.1097/HCO.0b013e32833f02fb 20885316

[48] Lamy A Devereaux PJ Prabhakaran D Taggart DP Hu S Paolasso E Off-pump or on-pump coronary-artery bypass grafting at 30 days. N Engl J Med. (2012) 366(16):1489–97. 10.1056/NEJMoa1200388 22449296

[49] Halkos ME Vassiliades TA Douglas JS Morris DC Rab ST Liberman HA Hybrid coronary revascularization versus off-pump coronary artery bypass grafting for the treatment of multivessel coronary artery disease. Ann Thorac Surg. (2011) 92(5):1695–702. 10.1016/j.athoracsur.2011.05.090 21939958

[50] Head SJ Milojevic M Taggart DP Puskas JD. Current practice of state-of-the-art surgical coronary revascularization. Circulation. (2017) 136(14):1331–45. 10.1161/CIRCULATIONAHA.116.022572 28972063

[51] Alexander JH. Efficacy and safety of edifoligide, an E2F transcription factor decoy, for prevention of vein graft failure following coronary artery bypass graft surgery PREVENT IV: a randomized controlled trial. J Am Med Assoc. (2005) 294(19):2446. 10.1001/jama.294.19.2446 16287955

[52] Parang P Arora R. Coronary vein graft disease: pathogenesis and prevention. Can J Cardiol. (2009) 25(2):e57–62. 10.1016/S0828-282X(09)70486-6 19214303 PMC2691920

[53] Bangalore S Toklu B Patel N Feit F Stone GW. Newer-generation ultrathin strut drug-eluting stents versus older second-generation thicker strut drug-eluting stents for coronary artery disease: meta-analysis of randomized trials. Circulation. (2018) 138(20):2216–26. 10.1161/CIRCULATIONAHA.118.034456 29945934

[54] Gąsior M Zembala MO Tajstra M Filipiak K Gierlotka M Hrapkowicz T Hybrid revascularization for multivessel coronary artery disease. JACC: Cardiovasc Interv. (2014) 7(11):1277–83. 25459040 10.1016/j.jcin.2014.05.025

[55] Adams C Burns DJP Chu MWA Jones PM Shridar K Teefy P Single-stage hybrid coronary revascularization with long-term follow-up. Eur J Cardiothorac Surg. (2014) 45(3):438–43. 10.1093/ejcts/ezt390 23956269

[56] Shen L Hu S Wang H Xiong H Zheng Z Li L One-stop hybrid coronary revascularization versus coronary artery bypass grafting and percutaneous coronary intervention for the treatment of multivessel coronary artery disease: 3-year follow-up results from a single institution. J Am Coll Cardiol. (2013) 61(25):2525–33. 10.1016/j.jacc.2013.04.007 23623906

[57] Lowenstern A Wu J Bradley SM Fanaroff AC Tcheng JE Wang TY. Current landscape of hybrid revascularization: a report from the NCDR CathPCI registry. Am Heart J. (2019) 215:167–77. 10.1016/j.ahj.2019.06.014 31349108 PMC6753051

[58] Panoulas VF Colombo A Margonato A Maisano F. Hybrid coronary revascularization: promising, but yet to take off. J Am Coll Cardiol. (2015) 65(1):85–97. 10.1016/j.jacc.2014.04.093 25572514

[59] Kiaii B McClure RS Stewart P Rayman R Swinamer SA Suematsu Y Simultaneous integrated coronary artery revascularization with long-term angiographic follow-up. J Thorac Cardiovasc Surg. (2008) 136(3):702–8. 10.1016/j.jtcvs.2008.02.081 18805275

[60] Reicher B Poston RS Mehra MR Joshi A Odonkor P Kon Z Simultaneous “hybrid” percutaneous coronary intervention and minimally invasive surgical bypass grafting: feasibility, safety, and clinical outcomes. Am Heart J. (2008) 155(4):661–7. 10.1016/j.ahj.2007.12.032 18371473 PMC2636970

[61] Bonatti JO Zimrin D Lehr EJ Vesely M Kon ZN Wehman B Hybrid coronary revascularization using robotic totally endoscopic surgery: perioperative outcomes and 5-year results. Ann Thorac Surg. (2012) 94(6):1920–6. 10.1016/j.athoracsur.2012.05.041 23103003

[62] Halkos ME Walker PF Vassiliades TA Douglas JS Devireddy C Guyton RA Clinical and angiographic results after hybrid coronary revascularization. Ann Thorac Surg. (2014) 97(2):484–90. 10.1016/j.athoracsur.2013.08.041 24140212

[63] Puskas JD Halkos ME DeRose JJ Bagiella E Miller MA Overbey J Hybrid coronary revascularization for the treatment of multivessel coronary artery disease a multicenter observational study. J Am Coll Cardiol. (2016) 68(4):356–65. 10.1016/j.jacc.2016.05.032 27443431 PMC5142519

[64] O’Fee K Deych E Ciani O Brown DL. Assessment of nonfatal myocardial infarction as a surrogate for all-cause and cardiovascular mortality in treatment or prevention of coronary artery disease: a meta-analysis of randomized clinical trials. JAMA Intern Med. (2021) 181(12):1575–87. 10.1001/jamainternmed.2021.5726 34694318 PMC8546625

[65] Stone GW Maehara A Muller JE Plaque characterization to inform the prediction and prevention of periprocedural myocardial infarction during percutaneous coronary intervention the CANARY trial (coronary assessment by near-infrared of atherosclerotic rupture-prone yellow). JACC: Cardiovasc Interv. (2015) 8(7):927–36. 26003018 10.1016/j.jcin.2015.01.032

[66] Park D-W Kim Y-H Yun S-C Ahn J-M Lee J-Y Kim W-J Impact of the angiographic mechanisms underlying periprocedural myocardial infarction after drug-eluting stent implantation. Am J Cardiol. (2014) 113(7):1105–10. 10.1016/j.amjcard.2013.12.016 24513476

[67] Baumgarten H Rolf A Weferling M Graessle T Fischer-Rasokat U Keller T Outcomes after early postoperative myocardial infarction due to graft failure in patients undergoing coronary artery bypass grafting. J Inv Cardiol. (2023) 35(4):E161–8. 10.25270/jic/21.00376 36827082

[68] Thielmann M Sharma V Al-Attar N Bulluck H Bisleri G Bunge JJ ESC joint working groups on cardiovascular surgery and the cellular biology of the heart position paper: peri-operativemyocardial injury and infarction in patients undergoing coronary artery bypass graft surgery. Eur Heart J. (2017) 38(31):2392–411. 10.1093/eurheartj/ehx383 28821170 PMC5808635

[69] Heuts S Gollmann-Tepeköylü C Denessen EJS Olsthoorn JR Romeo JLR Maessen JG Cardiac troponin release following coronary artery bypass grafting: mechanisms and clinical implications. Eur Heart J. (2023) 44(2):100–12. 10.1093/eurheartj/ehac604 36337034 PMC9897191

[70] Thygesen K Alpert JS Jaffe AS Chaitman BR Bax JJ Morrow DA Fourth universal definition of myocardial infarction (2018). Circulation. (2018) 138(20):e618–51. 10.1161/CIR.0000000000000617 30571511

[71] Bulluck H Paradies V Barbato E Baumbach A Bøtker HE Capodanno D Prognostically relevant periprocedural myocardial injury and infarction associated with percutaneous coronary interventions: a consensus document of the ESC working group on cellular biology of the heart and European association of percutaneous cardiovascular interventions (EAPCI). Eur Heart J. (2021) 42(27):2630–2642C. 10.1093/eurheartj/ehab271 34059914 PMC8282317

[72] Ofori-Asenso R Zomer E Chin KL Markey P Si S Ademi Z Prevalence and impact of non-cardiovascular comorbidities among older adults hospitalized for non-ST segment elevation acute coronary syndrome. Cardiovasc Diagn Ther. (2019) 9(3):250–61. 10.21037/cdt.2019.04.06 31275815 PMC6603501

[73] Silvain J Zeitouni M Paradies V Zheng HL Ndrepepa G Cavallini C Cardiac procedural myocardial injury, infarction, and mortality in patients undergoing elective percutaneous coronary intervention: a pooled analysis of patient-level data. Eur Heart J. (2021) 42(4):323–34. 10.1093/eurheartj/ehaa885 33257958 PMC7850039

[74] Devereaux PJ Lamy A Chan MTV Allard RV Lomivorotov VV Landoni G High-sensitivity troponin I after cardiac surgery and 30-day mortality. N Engl J Med. (2022) 386(9):827–36. 10.1056/NEJMoa2000803 35235725

[75] Hara H Serruys PW Takahashi K Kawashima H Ono M Gao C Impact of peri-procedural myocardial infarction on outcomes after revascularization. J Am Coll Cardiol. (2020) 76(14):1622–39. 10.1016/j.jacc.2020.08.009 33004127

[76] Gregson J Stone GW Ben-Yehuda O Redfors B Kandzari DE Morice M-C Implications of alternative definitions of peri-procedural myocardial infarction after coronary revascularization. J Am Coll Cardiol. (2020) 76(14):1609–21. 10.1016/j.jacc.2020.08.016 33004126

[77] Gaudino M Di Franco A Dimagli A Biondi-Zoccai G Rahouma M Perezgrovas Olaria R Correlation between periprocedural myocardial infarction, mortality, and quality of life in coronary revascularization trials: a meta-analysis. J Soc Cardiovasc Angiogr Interv. (2023) 2(3):100591. 10.1016/j.jscai.2023.100591 39130713 PMC11307952

[78] Armillotta M Bergamaschi L Paolisso P Belmonte M Angeli F Sansonetti A Prognostic relevance of type 4A myocardial infarction and periprocedural myocardial injury in patients with non–ST-segment–elevation myocardial infarction. Circulation. (2025) 151(11):760–72. 10.1161/CIRCULATIONAHA.124.070729 39968630 PMC11913249

[79] Crea F Libby P. Acute coronary syndromes: the way forward from mechanisms to precision treatment. Circulation. (2017) 136(12):1155–66. 10.1161/CIRCULATIONAHA.117.029870 28923905 PMC5679086

[80] Lee T Murai T Isobe M Kakuta T. Impact of coronary plaque morphology assessed by optical coherence tomography on cardiac troponin elevation in patients with non-ST segment elevation acute coronary syndrome. Catheter Cardiovasc Interv. (2017) 90(6):905–14. 10.1002/ccd.27037 28303685

[81] Paolisso P Foà A Bergamaschi L Donati F Fabrizio M Chiti C Hyperglycemia, inflammatory response and infarct size in obstructive acute myocardial infarction and MINOCA. Cardiovasc Diabetol. (2021) 20(1):33. 10.1186/s12933-021-01222-9 33530978 PMC7856791

[82] Ebrahimi R Dyke C Mehran R Manoukian SV Feit F Cox DA Outcomes following Pre-operative clopidogrel administration in patients with acute coronary syndromes undergoing coronary artery bypass surgery. The ACUITY (acute catheterization and urgent intervention triage strategY) trial. J Am Coll Cardiol. (2009) 53(21):1965–72. 10.1016/j.jacc.2009.03.006 19460609

[83] Dworeck C Redfors B Angerås O Haraldsson I Odenstedt J Ioanes D Association of pretreatment with P2Y12 receptor antagonists preceding percutaneous coronary intervention in non-ST-segment elevation acute coronary syndromes with outcomes. JAMA Netw Open. (2020) 3(10):e2018735–e2018735. 10.1001/jamanetworkopen.2020.18735 33001202 PMC7530628

[84] Schüpke S Neumann F-J Menichelli M Mayer K Bernlochner I Wöhrle J Ticagrelor or prasugrel in patients with acute coronary syndromes. N Engl J Med. (2019) 381(16):1524–34. 10.1056/NEJMoa1908973 31475799

[85] Montalescot G Bolognese L Dudek D Goldstein P Hamm C Tanguay J-F Pretreatment with prasugrel in non–ST-segment elevation acute coronary syndromes. N Engl J Med. (2013) 369(11):999–1010. 10.1056/NEJMoa1308075 23991622

[86] Montalescot G Collet JP Ecollan P Bolognese L Ten Berg J Dudek D Effect of prasugrel pre-treatment strategy in patients undergoing percutaneous coronary intervention for NSTEMI: the ACCOAST-PCI study. J Am Coll Cardiol. (2014) 64(24):2563–71. 10.1016/j.jacc.2014.08.053 25524333

[87] Tarantini G Mojoli M Varbella F Caporale R Rigattieri S Ando G Timing of oral P2Y12 inhibitor administration in patients with non-ST-segment elevation acute coronary syndrome. J Am Coll Cardiol. (2020) 76(21):2450–9. 10.1016/j.jacc.2020.08.053 32882390

[88] Clopidogrel in Unstable Angina to Prevent Recurrent Events Trial Investigators. Effects of clopidogrel in addition to aspirin in patients with acute coronary syndromes without ST-segment elevation. N Engl J Med. (2001) 345(7):494–502. 10.1056/NEJMoa010746 11519503

[89] Silvain J Rakowski T Lattuca B Liu Z Bolognese L Goldstein P Interval from initiation of prasugrel to coronary angiography in patients with non–ST-segment elevation myocardial infarction. J Am Coll Cardiol. (2019) 73(8):906–14. 10.1016/j.jacc.2018.11.055 30819358

[90] Harrington RA Stone GW McNulty S White HD Lincoff AM Gibson CM Platelet inhibition with cangrelor in patients undergoing PCI. N Engl J Med. (2009) 361(24):2318–29. 10.1056/NEJMoa0908628 19915221

[91] Bhatt DL Lincoff AM Gibson CM Stone GW McNulty S Montalescot G Intravenous platelet blockade with cangrelor during PCI. N Engl J Med. (2009) 361(24):2330–41. 10.1056/NEJMoa0908629 19915222

[92] Abtan J Steg PG Stone GW Mahaffey KW Gibson CM Hamm CW Efficacy and safety of cangrelor in preventing periprocedural complications in patients with stable angina and acute coronary syndromes undergoing percutaneous coronary intervention the CHAMPION PHOENIX trial. JACC Cardiovasc Interv. (2016) 9(18):1905–13. 10.1016/j.jcin.2016.06.046 27659566

[93] Steg PG Bhatt DL Hamm CW Stone GW Gibson CM Mahaffey KW Effect of cangrelor on periprocedural outcomes in percutaneous coronary interventions: a pooled analysis of patient-level data. Lancet. (2013) 382(9909):1981–92. 10.1016/S0140-6736(13)61615-3 24011551

[94] Mehta RH Roe MT Mulgund J Ohman EM Cannon CP Gibler WB Acute clopidogrel use and outcomes in patients with non-ST-segment elevation acute coronary syndromes undergoing coronary artery bypass surgery. J Am Coll Cardiol. (2006) 48(2):281–6. 10.1016/j.jacc.2006.04.029 16843176

[95] Berger JS Frye CB Harshaw Q Edwards FH Steinhubl SR Becker RC. Impact of clopidogrel in patients with acute coronary syndromes requiring coronary artery bypass surgery. A multicenter analysis. J Am Coll Cardiol. (2008) 52(21):1693–701. 10.1016/j.jacc.2008.08.031 19007688

[96] Rojas SV Trinh-Adams ML Uribarri A Fleissner F Iablonskii P Rojas-Hernandez S Early surgical myocardial revascularization in non-ST-segment elevation acute coronary syndrome. J Thorac Dis. (2019) 11(11):4444–52. 10.21037/jtd.2019.11.08 31903232 PMC6940209

[97] Bianco V Kilic A Gleason TG Aranda-Michel E Wang Y Navid F Timing of coronary artery bypass grafting after acute myocardial infarction may not influence mortality and readmissions. J Thorac Cardiovasc Surg. (2021) 161(6):2056–2064.e4. 10.1016/j.jtcvs.2019.11.061 31952832

[98] Weiss ES Chang DD Joyce DL Nwakanma LU Yuh DD. Optimal timing of coronary artery bypass after acute myocardial infarction: a review of California discharge data. J Thorac Cardiovasc Surg. (2008) 135(3):503–11. 10.1016/j.jtcvs.2007.10.042 18329460

[99] Hadaya J Sanaiha Y Tran Z Downey P Shemin RJ Benharash P. Timing of coronary artery bypass grafting in acute coronary syndrome: a national analysis. Ann Thorac Surg. (2022) 113(5):1482–90. 10.1016/j.athoracsur.2021.05.057 34126075

[100] Gaudino M Angelini GD Antoniades C Bakaeen F Benedetto U Calafiore AM Off-pump coronary artery bypass grafting: 30 years of debate. J Am Heart Assoc. (2018) 7(16):e009934. 10.1161/JAHA.118.009934 30369328 PMC6201399

[101] Kawamoto S Miyata H Motomura N Tanemoto K Takamoto S Saiki Y. Surgical outcomes of isolated coronary artery bypass grafting for acute coronary syndrome: based on the Japan adult cardiovascular surgery database. Circ J. (2018) 82(1):123–30. 10.1253/circj.CJ-17-0561 28867682

[102] Hwang B Williams ML Tian DH Yan TD Misfeld M. Coronary artery bypass surgery for acute coronary syndrome: a network meta-analysis of on-pump cardioplegic arrest, off-pump, and on-pump beating heart strategies. J Card Surg. (2022) 37(12):5290–9. 10.1111/jocs.17149 36349729 PMC10099567

[103] Ntinopoulos V Haeussler A Odavic D Papadopoulos N Rings L Dushaj S Conversion from off-pump to on-pump coronary artery bypass grafting: impact of surgeon and anaesthetist experience. Interdiscip Cardiovasc Thorac Surg. (2023) 37(6):ivad205. 10.1093/icvts/ivad205 38123498 PMC10751234

[104] Keeling B Thourani V Aliawadi G Kim S Cyr D Badhwar V Conversion from off-pump coronary artery bypass grafting to on-pump coronary artery bypass grafting. Ann Thorac Surg. (2017) 104(4):1267–74. 10.1016/j.athoracsur.2017.03.032 28610886

[105] Tariq K Zia K Mangi A Amanullah M Chaudry PA Karim M. Conversion from off to on-pump coronary artery bypass grafting. Is it avoidable? Cureus. (2020) 12:e6791. 32140350 10.7759/cureus.6791 PMC7046009

[106] Puskas JD Williams WH O'Donnell R Patterson RE Sigman SR Smith AS Off-pump and on-pump coronary artery bypass grafting are associated with similar graft patency, myocardial ischemia, and freedom from reintervention: long-term follow-up of a randomized trial. Ann Thorac Surg. (2011) 91(6):1836–43. 10.1016/j.athoracsur.2010.12.043 21619980

[107] Harling L Moscarelli M Kidher E Fattouch K Ashrafian H Athanasiou T. The effect of off-pump coronary artery bypass on mortality after acute coronary syndrome: a meta-analysis. Int J Cardiol. (2013) 169(5):339–48. 10.1016/j.ijcard.2013.09.003 24161532

